# Supplementary material for: Vocational Interventions to Improve Employment Participation of People with Psychosocial Disability, Autism and/or Intellectual Disability: A Systematic Review
Source: Int J Environ Res Public Health. 2021 Nov 17;18(22):12083. doi: 10.3390/ijerph182212083 (PMC8618542; doi:10.3390/ijerph182212083)
Supplement: Supplementary file 1 [file ijerph-18-12083-s001.zip › Supplementary File S2_ID non randomized info.pdf]

Supplementary File S2: Non-randomized studies including participants with autism and intellectual disabilities

As we did not identify any RCTs including people with intellectual disabilities, and only three studies focusing on people with autism, we performed an additional systematic search for non-randomized interventions for these populations of interest.

Methods

All searches are included below, following the PRISMA flowchart. The screening and extraction process remained the same for non-randomized studies as for the RCTs. After the removal of duplicates, two reviewers (including author HD) independently screened titles and abstracts using Covidence. Potentially relevant articles were screened in full in Covidence by HD, CM, MS.

For non-randomized studies, we planned to use the Joanna Briggs Institute (JBI) Critical Appraisal Checklist for Cohort studies for cohort studies and the JBI Critical Appraisal Checklist for Quasi-Experimental Studies for quasi-experimental studies.

Results

Figure S1: below shows the flow of non-randomized studies into this supplementary review. The titles and abstracts of 688 articles were screened, resulting in the full-text screening of eight articles. Following full-text screening, only two studies were eligible for inclusion, hence we did not synthesize results. Nonetheless a summary of these two studies is provided below [1, 2].

Kaya, C., Chan, F., Rumrill, P., Hartman, E., Wehman, P., Iwanga, K., Pai, C.H., Avellone, L. 2016. *Vocational rehabilitation services and competitive employment for transition-age youth with autism spectrum disorders.* Jounrnal of Vocational Rehabilitation **45**(1): 73-83	Multivariate logistic regression analysis of the 2011 Rehabilitation Services Administration's Case Service Reports to examine the extent to which demographic characteristics, disability benefits and vocational rehabilitation (VR) services influence competitive employment outcomes for transition-age youth with autism. Authors concluded the number and type of VR services had more influence on competitive employment than did demographic variables or disability benefits.
Langi, FFG., Oberoi, A., Balcazar, FE., Awsumb, J. 2017. *Vocational rehabilitation of transition-age youth with disabilities: A propensity-score mathed study.*Journal of Occupational Rehabilitation **27** (1):15-23	A population-based study on n=4422 youth with disability. Selected youth were classified into either targeted secondary transition program (START) or non-START treatment group. Employment outcomes were compared using propensity-score matching procedures. Results suggested START were more effective than regular VR services.

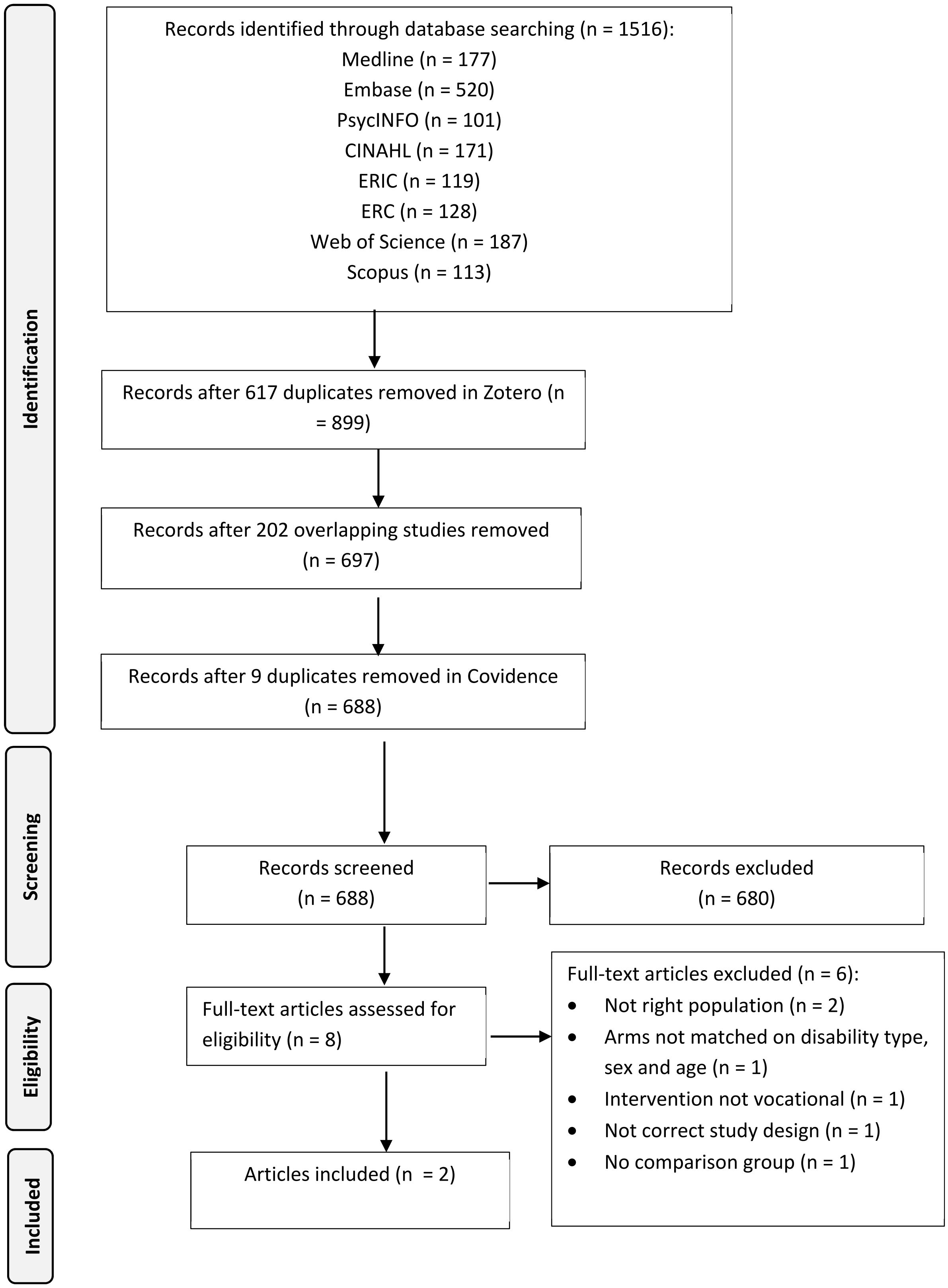

Figure S2: PRISMA flow diagram for non-randomised studies

Complete Search Strategy:
MEDLINE
1 randomized controlled trial.pt.
2 controlled clinical trial.pt.
3 randomi*ed.ab.
4 placebo.ab.
5 clinical trials as topic.sh.
6 randomly.ab.
7 trial.ti.
8 1 or 2 or 3 or 4 or 5 or 6 or 7
9 exp animals/ not humans.sh.
10 8 not 9[48]
11 exp Autism Spectrum Disorder/
12 ("autism spectrum disorder*" OR "autism" OR "autistic" OR "asperger*" OR "pervasive developmental disorder*").ti,ab.
13 11 or 12
14 exp intellectual disability/ or developmental disabilities/ or mentally disabled persons/
15 ("intellectual* disab*" OR "learning disab*" OR "developmental* disab*" OR "development* disorder*" OR "cognitive* disab*" OR "fragile x syndrome" OR "down* syndrome" OR "developmental delay*" OR "prader-willi syndrome" OR "fetal alcohol spectrum disorder*").ti,ab.
16 14 or 15
17 mental disorders/ or anxiety disorders/ or exp "bipolar and related disorders"/ or mood disorders/ or depressive disorder/ or depressive disorder, major/ or depressive disorder, treatment-resistant/ or "schizophrenia spectrum and other psychotic disorders"/ or affective disorders, psychotic/ or psychotic disorders/ or schizophrenia/ or mentally ill persons/
18 ("psychosocial* disab*" OR "severe mental illness*" OR "serious mental illness*" OR "severe mental health" OR "serious mental health" OR "psychiatric disorder*" OR "psychiatric illness*" OR "psychiatric condition*" OR "psychiatric* disab*" OR "schizophrenia" OR "schizoaffective" OR "schizo affective" OR "psychosis" OR "psychoses" OR "psychotic" OR "bipolar" OR ("affective disorder*" adj3 (severe or major)) OR ("depressi*" adj3 (severe or major)) OR ("anxiety" adj3 (severe or major))).ti,ab.
19 17 or 18
20 13 or 16 or 19
21 employment/ OR employment, supported/ OR sheltered workshops/ OR rehabilitation, vocational/ OR vocational education/ or vocational guidance/ or work/ or return to work/
22 ("employment" OR "employability" OR "employable" OR "project search" OR "ticket to work" OR "individual placement or support" OR "clubhouse" OR "sheltered work*" OR "disability enterprise*" OR "social enterprise*" OR "social firm*" OR "social cooperative*" OR "affirmative business*" OR "social purpose business*" or "vocational*" OR "prevocational" OR ((career or occupational) adj1 (guidance OR counse*ling OR plan* OR development)) OR "return to work" OR "work participation" OR "work read*" OR "work status" OR "work retention" OR apprenticeship* OR "work experience*" OR "occupational rehabilitation" OR "work rehabilitation" OR "school to work transition" OR "place and train" OR "place train" OR "train and place" OR "train place" OR "job coach*" OR "job interview*" OR "job search*" OR ((job OR work or workplace) adj4 (mentor* OR training or placement* OR program or programme))).ti,ab.
23 21 or 22
24 10 AND 20 AND 23
25 limit 24 to (english language and yr="2010 -Current") **[RCTs]**
26 cohort studies/ or follow-up studies/ or longitudinal studies/ or prospective studies/ or comparative study/ or controlled clinical trial/ or controlled before-after studies/
27 ("quasi-experiment*" OR "experimental study" OR "clinical trial" OR "control* trial" OR "control* study" OR "before and after study" OR "cohort study" OR "comparative study" OR "intervention study" OR "longitudinal study" OR "follow-up study" OR matched OR "prospective study").ti,ab.
28 (clinical trial OR comparative study OR controlled clinical trial).pt.
29 26 or 27 or 28

30 13 or 16
31 29 AND 30 AND 23
32 31 NOT 25
33 limit 32 to (english language and yr="2010 -Current") **[non-randomised studies only]**

Embase
1 (crossover* or "cross over*" or placebo* or (doubl* adj blind*) or allocat* or random*).ti,ab,ot.
2 trial.ti.
3 crossover-procedure/ or double-blind procedure/ or single-blind procedure/ or randomized controlled trial/
4 1 or 2 or 3[48]
5 exp autism/ or ("autism spectrum disorder*" OR "autism" OR "autistic" OR "asperger*" OR "pervasive developmental disorder*").ti,ab.
6 intellectual impairment/ or mental deficiency/ or down syndrome/ or x linked mental retardation/ or developmental disorder/ or mentally disabled persons/ or ("intellectual* disab*" OR "learning disab*" OR "developmental* disab*" OR "development* disorder*" OR "cognitive* disab*" OR "fragile x syndrome" OR "down* syndrome" OR "developmental delay*" OR "prader-willi syndrome" OR "fetal alcohol spectrum disorder*").ti,ab.
7 mental disease/ or anxiety disorder/ or mood disorder/ or major affective disorder/ or affective psychosis/ or psychosis/ or schizophrenia spectrum disorder/ or schizoaffective psychosis/ or bipolar disorder/ or ("psychosocial* disab*" OR "severe mental illness*" OR "serious mental illness*" OR "severe mental health" OR "serious mental health" OR "psychiatric disorder*" OR "psychiatric illness*" OR "psychiatric condition*" OR "psychiatric* disab*" OR "schizophrenia" OR "schizoaffective" OR "schizo affective" OR "psychosis" OR "psychoses" OR "psychotic" OR "bipolar" OR ("affective disorder*" adj3 (severe or major)) OR ("depressi*" adj3 (severe or major)) OR ("anxiety" adj3 (severe or major))).ti,ab.
8 5 or 6 or 7
9 employment/ or employment status/ or self-employment/ or supported employment/ or sheltered workshop/ or permanent employment/ or temporary employment/ or full time employment/ or parttime employment/ or vocational rehabilitation/ or vocational education/ or vocational guidance/ or work capacity/ or return to work/ or work disability/ or work experience/ or work/ or work capacity/ or work environment/ or job accommodation/ or job experience/ or workplace/ or ("employment" OR "employability" OR "employable" OR "project search" OR "ticket to work" OR "individual placement or support" OR "clubhouse" OR "sheltered work*" OR "disability enterprise*" OR "social enterprise*" OR "social firm*" OR "social cooperative*" OR "affirmative business*" OR "social purpose business*" or "vocational*" OR "prevocational" OR ((career or occupational) adj1 (guidance OR counse*ling OR plan* OR development)) OR "return to work" OR "work participation" OR "work read*" OR "work status" OR "work retention" OR apprenticeship* OR "work experience*" OR "occupational rehabilitation" OR "work rehabilitation" OR "school to work transition" OR "place and train" OR "place train" OR "train and place" OR "train place" OR "job coach*" OR "job interview*" OR "job search*" OR ((job OR work or workplace) adj4 (mentor* OR training or placement* OR program or programme))).ti,ab.
10 4 and 8 and 9
11 limit 10 to (english language and yr="2010 -Current" and article) **[RCTs]**
12 cohort analysis/ or follow up/ or longitudinal study/ or prospective study/ or intervention study/ or comparative study/ or controlled study/ or controlled clinical trial/ or pretest posttest control group design/ or experimental study/ or quasi experimental study/
13 ("quasi-experiment*" OR "experimental study" OR "clinical trial" OR "control* trial" OR "control* study" OR "before and after study" OR "cohort study" OR "comparative study" OR "intervention study" OR "longitudinal study" OR "follow-up study" OR matched OR "prospective study").ti,ab.
14 (clinical trial OR controlled clinical trial).ct.
15 12 or 13 or 14
16 5 or 6
17 15 and 16 and 9
18 17 NOT 11

19 limit 18 to (english language and yr="2010 -Current" and article) **[non-randomised studies only]**

PsycINFO
1 placebo/
2 follow up studies/
3 placebo*.tw.
4 random*.tw.
5 comparative stud*.tw.
6 (clinical adj3 trial*).tw.
7 (research adj3 design).tw.
8 (evaluat* adj3 stud*).tw.
9 (prospectiv* adj3 stud*).tw.
10 ((singl* or doubl* or trebl* or tripl*) adj3 (blind* or mask*)).tw.
11 1 or 2 or 3 or 4 or 5 or 6 or 7 or 8 or 9 or 10[49]
12 autism spectrum disorders/
13 ("autism spectrum disorder*" OR "autism" OR "autistic" OR "asperger*" OR "pervasive developmental disorder*").ti,ab.
14 intellectual development disorder/ or down's syndrome/ or cognitive impairment/ or developmental disabilities/ or fetal alcohol syndrome/ or fragile x syndrome/ or prader willi syndrome/
15 ("intellectual* disab*" OR "learning disab*" OR "developmental* disab*" OR "development* disorder*" OR "cognitive* disab*" OR "fragile x syndrome" OR "down* syndrome" OR "developmental delay*" OR "prader-willi syndrome" OR "fetal alcohol spectrum disorder*").ti,ab.
16 mental disorders/ or affective disorders/ or anxiety disorders/ or bipolar disorder/ or chronic mental illness/ or psychosis/ or serious mental illness/ or major depression/ or affective psychosis/ or schizophrenia/ or schizoaffective disorder/
17 ("psychosocial* disab*" OR "severe mental illness*" OR "serious mental illness*" OR "severe mental health" OR "serious mental health" OR "psychiatric disorder*" OR "psychiatric illness*" OR "psychiatric condition*" OR "psychiatric* disab*" OR "schizophrenia" OR "schizoaffective" OR "schizo affective" OR "psychosis" OR "psychoses" OR "psychotic" OR "bipolar" OR ("affective disorder*" adj3 (severe or major)) OR ("depressi*" adj3 (severe or major)) OR ("anxiety" adj3 (severe or major))).ti,ab.
18 12 or 13 or 14 or 15 or 16 or 17
19 employment status/ or employability/ or reemployment/ or self-employment/ or employee skills/ or sheltered workshops/ or exp vocational rehabilitation/ or school to work transition/ or occupational guidance/ or exp vocational education/ or sheltered workshops/ or job search/ or job experience level/ or labor market/ or career development/ or job applicant interviews/ or occupational adjustment/
20 ("employment" OR "employability" OR "employable" OR "project search" OR "ticket to work" OR "individual placement or support" OR "clubhouse" OR "sheltered work*" OR "disability enterprise*" OR "social enterprise*" OR "social firm*" OR "social cooperative*" OR "affirmative business*" OR "social purpose business*" or "vocational*" OR "prevocational" OR ((career or occupational) adj1 (guidance OR counse*ling OR plan* OR development)) OR "return to work" OR "work participation" OR "work read*" OR "work status" OR "work retention" OR apprenticeship* OR "work experience*" OR "occupational rehabilitation" OR "work rehabilitation" OR "school to work transition" OR "place and train" OR "place train" OR "train and place" OR "train place" OR "job coach*" OR "job interview*" OR "job search*" OR ((job OR work or workplace) adj4 (mentor* OR training or placement* OR program or programme))).ti,ab.
21 19 or 20
22 11 AND 18 AND 21
23 limit 22 to (english language and journal article and yr="2010 -Current") **[RCTs]**
24 experimental design/ or clinical trials/ or cohort analysis/ or followup studies/ or longitudinal studies/ or experiment controls/ or experimental methods/ or quasi experimental methods/
25 ("quasi-experiment*" OR "experimental study" OR "clinical trial" OR "control* trial" OR "control* study" OR "before and after study" OR "cohort study" OR "comparative study" OR "intervention study" OR "longitudinal study" OR "follow-up study" OR matched OR "prospective study").ti,ab.

26 24 or 25
27 12 or 13 or 14 or 15
28 26 and 27 and 21
29 28 NOT 23
30 limit 29 to (english language and journal article and yr="2010 -Current") **[non-randomised studies only]**

Web of Science
1 TI= (randomi*ed)
2 AB= (randomi*ed)
3 AB= (placebo)
4 AB= (randomly)
5 TI= (trial)
6 1 or 2 or 3 or 4 or 5
7 TS=("autism spectrum disorder*" OR "autism" OR "autistic" OR "asperger*" OR "pervasive developmental disorder*")
8 TS=("intellectual* disab*" OR "learning disab*" OR "developmental* disab*" OR "development* disorder*" OR "cognitive* disab*" OR "fragile x syndrome" OR "down* syndrome" OR "developmental delay*" OR "prader-willi syndrome" OR "fetal alcohol spectrum disorder*")
9 TS=("psychosocial* disab*" OR "severe mental illness*" OR "serious mental illness*" OR "severe mental health" OR "serious mental health" OR "psychiatric disorder*" OR "psychiatric illness*" OR "psychiatric condition*" OR "psychiatric* disab*" OR "schizophrenia" OR "schizoaffective" OR "schizo affective" OR "psychosis" OR "psychoses" OR "psychotic" OR "bipolar" OR ("affective disorder*" NEAR/2 (severe or major)) OR ("depressi*" NEAR/2 (severe or major)) OR ("anxiety" NEAR/2 (severe or major)))
10 7 or 8 or 9
11 TS=("employment" OR "employability" OR "employable" OR "project search" OR "ticket to work" OR "individual placement or support" OR "clubhouse" OR "sheltered work*" OR "disability enterprise*" OR "social enterprise*" OR "social firm*" OR "social cooperative*" OR "affirmative business*" OR "social purpose business*" or "vocational*" OR "prevocational" OR ((career or occupational) NEAR/0 (guidance OR counse*ling OR plan* OR development)) OR "return to work" OR "work participation" OR "work read*" OR "work status" OR "work retention" OR apprenticeship* OR "work experience*" OR "occupational rehabilitation" OR "work rehabilitation" OR "school to work transition" OR "place and train" OR "place train" OR "train and place" OR "train place" OR "job coach*" OR "job interview*" OR "job search*" OR ((job OR work or workplace) NEAR/3 (mentor* OR training or placement* OR program or programme)))
12 6 and 10 and 11 AND LANGUAGE: (English) AND DOCUMENT TYPES: (Article)
Refined by: PUBLICATION YEARS: (2020 OR 2012 OR 2019 OR 2011 OR 2018 OR 2010 OR 2017 OR 2016 OR 2015 OR 2014 OR 2013) **[RCTs]**
13 TS= ("quasi-experiment*" OR "experimental study" OR "clinical trial" OR "control* trial" OR "control* study" OR "before and after study" OR "cohort study" OR "comparative study" OR "intervention study" OR "longitudinal study" OR "follow-up study" OR matched OR "prospective study")
14 7 or 8
15 13 and 14 and 11
16 15 NOT 12 AND LANGUAGE: (English) AND DOCUMENT TYPES: (Article)
Refined by: PUBLICATION YEARS: (2020 OR 2012 OR 2019 OR 2011 OR 2018 OR 2010 OR 2017 OR 2016 OR 2015 OR 2014 OR 2013) **[non-randomised studies only]**

SCOPUS
1 TITLE-ABS (randomi*ed)
2 ABS (placebo)
3 ABS (randomly)
4 TITLE (trial)
5 1 or 2 or 3 or 4
6 TITLE-ABS ("autism spectrum disorder*" OR "autism" OR "autistic" OR "asperger*" OR "pervasive developmental disorder*")

7 TITLE-ABS ("intellectual* disab*" OR "learning disab*" OR "developmental* disab*" OR "development* disorder*" OR "cognitive* disab*" OR "fragile x syndrome" OR "down* syndrome" OR "developmental delay*" OR "prader-willi syndrome" OR "fetal alcohol spectrum disorder*")
8 TITLE-ABS ("psychosocial* disab*" OR "severe mental illness*" OR "serious mental illness*" OR "severe mental health" OR "serious mental health" OR "psychiatric disorder*" OR "psychiatric illness*" OR "psychiatric condition*" OR "psychiatric* disab*" OR "schizophrenia" OR "schizoaffective" OR "schizo affective" OR "psychosis" OR "psychoses" OR "psychotic" OR "bipolar" OR ("affective disorder*" W/2 (severe or major)) OR ("depressi*" W/2 (severe or major)) OR ("anxiety" W/2 (severe or major)))
9 11 or 12 or 13
10 TITLE-ABS ("employment" OR "employability" OR "employable" OR "project search" OR "ticket to work" OR "individual placement or support" OR "clubhouse" OR "sheltered work*" OR "disability enterprise*" OR "social enterprise*" OR "social firm*" OR "social cooperative*" OR "affirmative business*" OR "social purpose business*" or "vocational*" OR "prevocational" OR ((career or occupational) W/0 (guidance OR counse*ling OR plan* OR development)) OR "return to work" OR "work participation" OR "work read*" OR "work status" OR "work retention" OR apprenticeship* OR "work experience*" OR "occupational rehabilitation" OR "work rehabilitation" OR "school to work transition" OR "place and train" OR "place train" OR "train and place" OR "train place" OR "job coach*" OR "job interview*" OR "job search*" OR ((job OR work or workplace) W/3 (mentor* OR training or placement* OR program or programme)))
11 5 AND 9 AND 10 AND (LIMIT-TO (PUBYEAR , 2020) OR LIMIT-TO (PUBYEAR , 2019) OR LIMIT-TO (PUBYEAR , 2018) OR LIMIT-TO (PUBYEAR , 2017) OR LIMIT-TO (PUBYEAR , 2016) OR LIMIT-TO (PUBYEAR , 2015) OR LIMIT-TO (PUBYEAR , 2014) OR LIMIT-TO (PUBYEAR , 2013) OR LIMIT-TO (PUBYEAR , 2012) OR LIMIT-TO (PUBYEAR , 2011) OR LIMIT-TO (PUBYEAR , 2010)) AND (LIMIT-TO (DOCTYPE , "ar")) AND (LIMIT-TO (LANGUAGE , "English")) **[RCTs]**
12 TITLE-ABS ("quasi-experiment*" OR "experimental study" OR "clinical trial" OR "control* trial" OR "control* study" OR "before and after study" OR "cohort study" OR "comparative study" OR "intervention study" OR "longitudinal study" OR "follow-up study" OR matched OR "prospective study")
13 6 or 7
14 12 AND 13 AND 10
15 14 NOT 11 AND (LIMIT-TO (PUBYEAR , 2020) OR LIMIT-TO (PUBYEAR , 2019) OR LIMIT-TO (PUBYEAR , 2018) OR LIMIT-TO (PUBYEAR , 2017) OR LIMIT-TO (PUBYEAR , 2016) OR LIMIT-TO (PUBYEAR , 2015) OR LIMIT-TO (PUBYEAR , 2014) OR LIMIT-TO (PUBYEAR , 2013) OR LIMIT-TO (PUBYEAR , 2012) OR LIMIT-TO (PUBYEAR , 2011) OR LIMIT-TO (PUBYEAR , 2010)) AND (LIMIT-TO (DOCTYPE , "ar")) AND (LIMIT-TO (LANGUAGE , "English")) **[non-randomised studies only]**

CINAHL
S1 MH randomized controlled trials
S2 MH double-blind studies
S3 MH single-blind studies
S4 MH random assignment
S5 MH pretest-posttest design
S6 MH cluster sample
S7 TI (randomised OR randomized)
S8 AB (random*)
S9 TI (trial)
S10 MH (sample size) AND AB (assigned OR allocated OR control)
S11 MH (placebos)
S12 PT (randomized controlled trial)
S13 AB (CONTROL W5 GROUP)
S14 MH (CROSSOVER DESIGN) OR MH (COMPARATIVE STUDIES)
S15 AB (CLUSTER W3 RCT)
S16 MH ANIMALS+
SS17 MH (ANIMAL STUDIES)
S18 TI (ANIMAL MODEL*)

S19 S16 OR S17 OR S18
S20 MH (HUMAN)
S21 S19 NOT S20
S22 S1 OR S2 OR S3 OR S4 OR S5 OR S6 OR S7 OR S8 OR S9 OR S10 OR S11 OR S12 OR S13 OR S14 OR S15
S23 S22 NOT S21[50]
S24 (MH "Pervasive Developmental Disorder-Not Otherwise Specified") OR (MH "Asperger Syndrome") OR (MH "Autistic Disorder") OR (MH "Child Development Disorders, Pervasive")
S25 TI ("autism spectrum disorder*" OR "autism" OR "autistic" OR "asperger*" OR "pervasive developmental disorder*")
S26 AB ("autism spectrum disorder*" OR "autism" OR "autistic" OR "asperger*" OR "pervasive developmental disorder*")
S27 S24 or S25 or S26
S28 (MH "Developmental Disabilities") OR (MH "Intellectual Disability") OR (MH "Down Syndrome") OR (MH "Prader-Willi Syndrome") OR (MH "Mental Retardation, X-Linked") OR (MH "Fragile X Syndrome") OR (MH "Mentally Disabled Persons")
S29 TI ("intellectual* disab*" OR "learning disab*" OR "developmental* disab*" OR "development* disorder*" OR "cognitive* disab*" OR "fragile x syndrome" OR "down* syndrome" OR "developmental delay*" OR "prader-willi syndrome" OR "fetal alcohol spectrum disorder*")
S30 AB ("intellectual* disab*" OR "learning disab*" OR "developmental* disab*" OR "development* disorder*" OR "cognitive* disab*" OR "fragile x syndrome" OR "down* syndrome" OR "developmental delay*" OR "prader-willi syndrome" OR "fetal alcohol spectrum disorder*")
S31 S28 or S29 or S30
S32 (MH "Mental Disorders") OR (MH "Mental Disorders, Chronic") OR (MH "Psychotic Disorders") OR (MH "Schizophrenia") OR (MH "Schizoaffective Disorder") OR (MH "Affective Disorders, Psychotic") OR (MH "Bipolar Disorder") OR (MH "Personality Disorders") OR (MH "Neurotic Disorders") OR (MH "Anxiety Disorders") OR (MH "Affective Disorders") OR (MH "Depression") OR (MH "Stress Disorders, Post-Traumatic")
S33 TI ("psychosocial* disab*" OR "severe mental illness*" OR "serious mental illness*" OR "severe mental health" OR "serious mental health" OR "psychiatric disorder*" OR "psychiatric illness*" OR "psychiatric condition*" OR "psychiatric* disab*" OR "schizophrenia" OR "schizoaffective" OR "schizo affective" OR "psychosis" OR "psychoses" OR "psychotic" OR "bipolar" OR ("affective disorder*" N2 (severe or major)) OR ("depressi*" N2 (severe or major)) OR ("anxiety" N2 (severe or major)))
S34 AB ("psychosocial* disab*" OR "severe mental illness*" OR "serious mental illness*" OR "severe mental health" OR "serious mental health" OR "psychiatric disorder*" OR "psychiatric illness*" OR "psychiatric condition*" OR "psychiatric* disab*" OR "schizophrenia" OR "schizoaffective" OR "schizo affective" OR "psychosis" OR "psychoses" OR "psychotic" OR "bipolar" OR ("affective disorder*" N2 (severe or major)) OR ("depressi*" N2 (severe or major)) OR ("anxiety" N2 (severe or major)))
S35 S32 or S33 or S34
S36 S27 or S31 or S35
S37 (MH "Employment") (MH "Job Interviews") OR (MH "Job Re-Entry") OR (MH "Employment Status") OR (MH "Self Employment") OR MH "Employment of Disabled+") OR (MH "Vocational Education") OR (MH "Rehabilitation, Vocational") OR (MH "Vocational Guidance") OR (MH "Sheltered Workshops") OR (MH "Job Experience") OR (MH "Work")
S38 TI ("employment" OR "employability" OR "employable" OR "project search" OR "ticket to work" OR "individual placement or support" OR "clubhouse" OR "sheltered work*" OR "disability enterprise*" OR "social enterprise*" OR "social firm*" OR "social cooperative*" OR "affirmative business*" OR "social purpose business*" or "vocational*" OR "prevocational" OR ((career or occupational) N0 (guidance OR counse*ling OR plan* OR development)) OR "return to work" OR "work participation" OR "work read*" OR "work status" OR "work retention" OR apprenticeship* OR "work experience*" OR "occupational rehabilitation" OR "work rehabilitation" OR "school to work transition" OR "place and train" OR "place train" OR "train and place" OR "train place" OR "job coach*" OR "job interview*" OR "job search*" OR ((job OR work or workplace) N3 (mentor* OR training or placement* OR program or programme)))
S39 AB ("employment" OR "employability" OR "employable" OR "project search" OR "ticket to work" OR "individual placement or support" OR "clubhouse" OR "sheltered work*" OR "disability enterprise*" OR "social

enterprise*" OR "social firm*" OR "social cooperative*" OR "affirmative business*" OR "social purpose business*" or "vocational*" OR "prevocational" OR ((career or occupational) N0 (guidance OR counse*ling OR plan* OR development)) OR "return to work" OR "work participation" OR "work read*" OR "work status" OR "work retention" OR apprenticeship* OR "work experience*" OR "occupational rehabilitation" OR "work rehabilitation" OR "school to work transition" OR "place and train" OR "place train" OR "train and place" OR "train place" OR "job coach*" OR "job interview*" OR "job search*" OR ((job OR work or workplace) N3 (mentor* OR training or placement* OR program or programme)))
S40 S37 or S38 or S39
S41 S23 AND S36 AND S40
Limiters - Published Date: 20100101-; English Language **[RCTs]**
S42 (MH "Prospective Studies+") OR (MH "Quasi-Experimental Studies+") OR (MH "Crossover Design") OR (MH "Experimental Studies") OR (MH "Clinical Trials") OR (MH "Controlled Before-After Studies") OR (MH "Pretest-Posttest Design+") OR (MH "Nonrandomized Trials") OR (MH "Intervention Trials")
S43 TI ("quasi-experiment*" OR "experimental study" OR "clinical trial" OR "control* trial" OR "control* study" OR "before and after study" OR "cohort study" OR "comparative study" OR "intervention study" OR "longitudinal study" OR "follow-up study" OR matched OR "prospective study")
S44 AB ("quasi-experiment*" OR "experimental study" OR "clinical trial" OR "control* trial" OR "control* study" OR "before and after study" OR "cohort study" OR "comparative study" OR "intervention study" OR "longitudinal study" OR "follow-up study" OR matched OR "prospective study")
S45 PT ("Clinical Trial")
S46 S42 or S43 or S44 or S45
S47 S27 or S31
S48 S46 AND S47 AND S40
Limiters - Published Date: 20100101-; English Language **[non-randomised studies only]**

ERIC
Limiters - Publication Type: Journal Articles
Limiters - Date Published: 2010-2020
S1 DE "Randomized Controlled Trials"
S2 ti (randomi*ed)
S3 ab (randomi*ed)
S4 ab (placebo)
S5 ab (randomly)
S6 ti (trial)
S7 S1 or S2 or S3 or S4 or S5 or S6
S8 DE ("Pervasive Developmental Disorders" OR "Asperger Syndrome" OR "Autism")
S9 TI ("autism spectrum disorder*" OR "autism" OR "autistic" OR "asperger*" OR "pervasive developmental disorder*")
S10 AB ("autism spectrum disorder*" OR "autism" OR "autistic" OR "asperger*" OR "pervasive developmental disorder*")
S11 S8 or S9 or S10
S12 DE ("Intellectual Disability" OR "Down Syndrome" OR "Mild Intellectual Disability" OR "Moderate Intellectual Disability" OR "Severe Intellectual Disability" OR "Developmental Disabilities" OR "Fetal Alcohol Syndrome")
S13 TI ("intellectual* disab*" OR "learning disab*" OR "developmental* disab*" OR "development* disorder*" OR "cognitive* disab*" OR "fragile x syndrome" OR "down* syndrome" OR "developmental delay*" OR "prader-willi syndrome" OR "fetal alcohol spectrum disorder*")
S14 AB ("intellectual* disab*" OR "learning disab*" OR "developmental* disab*" OR "development* disorder*" OR "cognitive* disab*" OR "fragile x syndrome" OR "down* syndrome" OR "developmental delay*" OR "prader-willi syndrome" OR "fetal alcohol spectrum disorder*")
S15 S12 or S13 or S14
S16 DE ("Mental Disorders" OR "Anxiety Disorders" OR "Psychosis" OR "Schizophrenia" OR "Depression (Psychology)" OR "Anxiety")

S17 TI ("psychosocial* disab*" OR "severe mental illness*" OR "serious mental illness*" OR "severe mental health" OR "serious mental health" OR "psychiatric disorder*" OR "psychiatric illness*" OR "psychiatric condition*" OR "psychiatric* disab*" OR "schizophrenia" OR "schizoaffective" OR "schizo affective" OR "psychosis" OR "psychoses" OR "psychotic" OR "bipolar" OR ("affective disorder*" N2 (severe or major)) OR ("depressi*" N2 (severe or major)) OR ("anxiety" N2 (severe or major)))
S18 AB ("psychosocial* disab*" OR "severe mental illness*" OR "serious mental illness*" OR "severe mental health" OR "serious mental health" OR "psychiatric disorder*" OR "psychiatric illness*" OR "psychiatric condition*" OR "psychiatric* disab*" OR "schizophrenia" OR "schizoaffective" OR "schizo affective" OR "psychosis" OR "psychoses" OR "psychotic" OR "bipolar" OR ("affective disorder*" N2 (severe or major)) OR ("depressi*" N2 (severe or major)) OR ("anxiety" N2 (severe or major)))
S19 S16 or S17 or S18
S20 S11 or S15 or S19
S21 DE "Employment" OR DE "Self Employment" OR DE "Supported Employment" OR DE "Careers" OR DE "Employment Opportunities" OR DE "Equal Opportunities (Jobs)" OR DE "Employment Programs" OR DE "Employment Services" OR DE "Employment Experience" OR DE "Employment Interviews" OR DE "Employment Potential" OR DE "Job Applicants" OR DE "Job Application" OR DE "Job Skills" OR DE "Work Experience" OR DE "Job Development" OR DE "Job Search Methods" OR DE "Career Awareness" OR DE "Career Guidance" OR DE "Career Counseling" OR DE "Career Development" OR DE "Career Education" OR DE "Career Exploration" OR DE "Career Planning" OR DE "Workplace Learning" OR "Off the Job Training" OR DE "On the Job Training" OR DE "Apprenticeships" OR DE "Professional Development" OR DE "Staff Development" OR "Vocational Education" OR DE "Prevocational Education" OR DE "Job Placement" OR DE "Job Shadowing" OR DE "Job Training" OR DE "Vocational Adjustment" OR DE "Vocational Rehabilitation" OR DE "Vocational Training Centers" OR DE "Work Experience Programs" OR DE "Sheltered Workshops" OR DE "Employment Patterns" OR DE "Work Attitudes" OR DE "Work Environment" or DE "Employees" OR DE "Quality of Working Life"
S22 TI ("employment" OR "employability" OR "employable" OR "project search" OR "ticket to work" OR "individual placement or support" OR "clubhouse" OR "sheltered work*" OR "disability enterprise*" OR "social enterprise*" OR "social firm*" OR "social cooperative*" OR "affirmative business*" OR "social purpose business*" or "vocational*" OR "prevocational" OR ((career or occupational) N0 (guidance OR counse*ling OR plan* OR development)) OR "return to work" OR "work participation" OR "work read*" OR "work status" OR "work retention" OR apprenticeship* OR "work experience*" OR "occupational rehabilitation" OR "work rehabilitation" OR "school to work transition" OR "place and train" OR "place train" OR "train and place" OR "train place" OR "job coach*" OR "job interview*" OR "job search*" OR ((job OR work or workplace) N3 (mentor* OR training or placement* OR program or programme)))
S23 AB ("employment" OR "employability" OR "employable" OR "project search" OR "ticket to work" OR "individual placement or support" OR "clubhouse" OR "sheltered work*" OR "disability enterprise*" OR "social enterprise*" OR "social firm*" OR "social cooperative*" OR "affirmative business*" OR "social purpose business*" or "vocational*" OR "prevocational" OR ((career or occupational) N0 (guidance OR counse*ling OR plan* OR development)) OR "return to work" OR "work participation" OR "work read*" OR "work status" OR "work retention" OR apprenticeship* OR "work experience*" OR "occupational rehabilitation" OR "work rehabilitation" OR "school to work transition" OR "place and train" OR "place train" OR "train and place" OR "train place" OR "job coach*" OR "job interview*" OR "job search*" OR ((job OR work or workplace) N3 (mentor* OR training or placement* OR program or programme)))
S24 S21 or S22 or S23
S29 S7 AND S20 AND S24
Limiters - Date Published: 20100101-; Language: English **[RCTs]**
S30 DE ("Longitudinal Studies" OR "Followup Studies" OR "Quasiexperimental Design" OR "Control Groups" OR "Experimental Groups" OR "Matched Groups" OR "Pretests Posttests")
S31 TI ("quasi-experiment*" OR "experimental study" OR "clinical trial" OR "control* trial" OR "control* study" OR "before and after study" OR "cohort study" OR "comparative study" OR "intervention study" OR "longitudinal study" OR "follow-up study" OR matched OR "prospective study")
S32 AB ("quasi-experiment*" OR "experimental study" OR "clinical trial" OR "control* trial" OR "control* study" OR "before and after study" OR "cohort study" OR "comparative study" OR "intervention study" OR "longitudinal study" OR "follow-up study" OR matched OR "prospective study")

S33 S30 or S31 or S32
S34 S11 or S15
S35 S33 and S34 and S24
S36 S35 NOT S29
Limiters - Date Published: 20100101-; Language: English **[non-randomised studies only]**

ERC
S1 TI (randomi*ed)
S2 AB (randomi*ed)
S3 AB (placebo)
S4 AB (randomly)
S5 TI (trial)
S6 S1 or S2 or S3 or S4 or S5
S7 DE "AUTISM" OR DE "AUTISM spectrum disorders" OR DE "ASPERGER'S syndrome"
S8 TI ("autism spectrum disorder*" OR "autism" OR "autistic" OR "asperger*" OR "pervasive developmental disorder*")
S9 AB ("autism spectrum disorder*" OR "autism" OR "autistic" OR "asperger*" OR "pervasive developmental disorder*")
S10 S7 or S8 or S9
S11 DE ("MENTAL disabilities" OR "DEVELOPMENTAL disabilities" OR "DOWN syndrome" OR "FETAL alcohol syndrome")
S12 TI ("intellectual* disab*" OR "learning disab*" OR "developmental* disab*" OR "development* disorder*" OR "cognitive* disab*" OR "fragile x syndrome" OR "down* syndrome" OR "developmental delay*" OR "prader-willi syndrome" OR "fetal alcohol spectrum disorder*")
S13 AB ("intellectual* disab*" OR "learning disab*" OR "developmental* disab*" OR "development* disorder*" OR "cognitive* disab*" OR "fragile x syndrome" OR "down* syndrome" OR "developmental delay*" OR "prader-willi syndrome" OR "fetal alcohol spectrum disorder*")
S14 S11 or S12 or S13
S15 DE ("Mental illness" OR "AFFECTIVE disorders" OR "ANXIETY" OR "MENTAL depression" OR "Schizophrenia" OR "Psychoses")
S16 TI ("psychosocial* disab*" OR "severe mental illness*" OR "serious mental illness*" OR "severe mental health" OR "serious mental health" OR "psychiatric disorder*" OR "psychiatric illness*" OR "psychiatric condition*" OR "psychiatric* disab*" OR "schizophrenia" OR "schizoaffective" OR "schizo affective" OR "psychosis" OR "psychoses" OR "psychotic" OR "bipolar" OR ("affective disorder*" N2 (severe or major)) OR ("depressi*" N2 (severe or major)) OR ("anxiety" N2 (severe or major)))
S17 AB ("psychosocial* disab*" OR "severe mental illness*" OR "serious mental illness*" OR "severe mental health" OR "serious mental health" OR "psychiatric disorder*" OR "psychiatric illness*" OR "psychiatric condition*" OR "psychiatric* disab*" OR "schizophrenia" OR "schizoaffective" OR "schizo affective" OR "psychosis" OR "psychoses" OR "psychotic" OR "bipolar" OR ("affective disorder*" N2 (severe or major)) OR ("depressi*" N2 (severe or major)) OR ("anxiety" N2 (severe or major)))
S18 S15 or S16 or S17
S19 S10 or S14 or S18
S20 DE ("EMPLOYMENT" OR "EMPLOYMENT & education" OR "SCHOOL-to-work transition" OR "WORK & education" OR "INDIVIDUALIZED transition plans" OR "VOCATIONAL guidance" OR "OCCUPATIONAL training" OR "VOCATIONAL education" OR "EMPLOYEE training" OR "INTERNSHIP programs" OR DE "CAREER development" OR "SUPPORTED employment" OR "EMPLOYMENT of people with disabilities" OR "VOCATIONAL rehabilitation" OR "SHELTERED workshops" OR "EMPLOYMENT agencies" OR "APPRENTICES" OR "APPRENTICESHIP programs" OR "INTERNSHIP programs" OR "JOB skills" OR "WORKPLACE literacy" OR "EMPLOYER-supported education")
S21 TI ("employment" OR "employability" OR "employable" OR "project search" OR "ticket to work" OR "individual placement or support" OR "clubhouse" OR "sheltered work*" OR "disability enterprise*" OR "social enterprise*" OR "social firm*" OR "social cooperative*" OR "affirmative business*" OR "social purpose business*" or "vocational*" OR "prevocational" OR ((career or occupational) N0 (guidance OR counse*ling OR plan* OR development)) OR "return to work" OR "work participation" OR "work read*" OR "work status" OR "work

retention" OR apprenticeship* OR "work experience*" OR "occupational rehabilitation" OR "work rehabilitation" OR "school to work transition" OR "place and train" OR "place train" OR "train and place" OR "train place" OR "job coach*" OR "job interview*" OR "job search*" OR ((job OR work or workplace) N3 (mentor* OR training or placement* OR program or programme)))
S22 AB ("employment" OR "employability" OR "employable" OR "project search" OR "ticket to work" OR "individual placement or support" OR "clubhouse" OR "sheltered work*" OR "disability enterprise*" OR "social enterprise*" OR "social firm*" OR "social cooperative*" OR "affirmative business*" OR "social purpose business*" or "vocational*" OR "prevocational" OR ((career or occupational) N0 (guidance OR counse*ling OR plan* OR development)) OR "return to work" OR "work participation" OR "work read*" OR "work status" OR "work retention" OR apprenticeship* OR "work experience*" OR "occupational rehabilitation" OR "work rehabilitation" OR "school to work transition" OR "place and train" OR "place train" OR "train and place" OR "train place" OR "job coach*" OR "job interview*" OR "job search*" OR ((job OR work or workplace) N3 (mentor* OR training or placement* OR program or programme)))
S23 S20 or S21 or S22
S24 S6 AND S19 AND S23
Limiters - Published Date: 20100101-; Language: English **[RCTs]**
S25 DE ("LONGITUDINAL method" OR "COHORT analysis" OR "COMPARATIVE studies" OR "EXPERIMENTAL design" OR "EXPERIMENTS" OR "CONTROL groups" OR "EXPERIMENTAL groups")
S26 TI ("quasi-experiment*" OR "experimental study" OR "clinical trial" OR "control* trial" OR "control* study" OR "before and after study" OR "cohort study" OR "comparative study" OR "intervention study" OR "longitudinal study" OR "follow-up study" OR matched OR "prospective study")
S27 AB ("quasi-experiment*" OR "experimental study" OR "clinical trial" OR "control* trial" OR "control* study" OR "before and after study" OR "cohort study" OR "comparative study" OR "intervention study" OR "longitudinal study" OR "follow-up study" OR matched OR "prospective study")
S28 S25 or S26 or S27
S29 S10 or S14
S30 S28 and S29 and S23
S31 S30 not S24
Limiters - Published Date: 20100101-; Language: English **[non-randomised studies only]**
