# Supplementary material for: Vocational Interventions to Improve Employment Participation of People with Psychosocial Disability, Autism and/or Intellectual Disability: A Systematic Review
Source: Int J Environ Res Public Health. 2021 Nov 17;18(22):12083. doi: 10.3390/ijerph182212083 (PMC8618542; doi:10.3390/ijerph182212083)
Supplement: Supplementary file 1 [file ijerph-18-12083-s001.zip › Supplementary File S4_Excluded studies_22_7_2021.pdf]

**Supplementary File S4**: Excluded studies and reasons for exclusion

| | Study | Reason for exclusion |
|---|---|---|
| 1 | Au, D.W.H.; Tsang, H.W.H.; So, W.W.Y.; Bell, M.D.; Cheung, V.; Yiu, M.G.C.; Tam, K.L.; Lee, G.T.H. 2014. Effects of integrated supported employment plus cognitive remediation training for people with schizophrenia and schizoaffective disorders. *Schizophrenia Research* 2014;166(1-3):297-303. | Medical, pharmacological and/or psychological interventions are part of comparator or control condition only |
| 2 | Baller, J.B.; Blyler, C.R.; Bronnikov, S.; Xie, H.; Bond, G.R.; Filion, K.; Hale, T. 2020. Long-term follow-up of a randomized trial of supported employment for SSDI beneficiaries with mental illness. *Psychiatric Services* 2020;71(3):243-249 | Not primary outcomes |
| 3 | Bejerholm, U.; Larsson, M.E.; Johanson, S. 2017. Supported employment adapted for people with affective disorders—A randomized controlled trial. *Journal of Affective Disorders* 2017;207():212-220 | Not people with autism, intellectual disability and/or psychosocial disability |
| 4 | Bell, M.D.; Choi, K.-H.; Dyer, C.; Wexler, B.E. 2014. Benefits of cognitive remediation and supported employment for schizophrenia patients with poor community functioning. *Psychiatric Services* 2014;65(4):469-475 | Medical, pharmacological and/or psychological interventions are part of comparator or control condition only |
| 5 | Bell, M.D.; Laws, H.; Pittman, B.; Johannesen, J.K. 2018. Comparison of focused cognitive training and portable "braingames" on functional outcomes for vocational rehabilitation participants. *Scientific Reports* 2018;8(1): | Intervention does not include vocational component |
| 6 | Boevink, W.; Kroon, H.; van Vugt, M.; Delespaul, P.; van Os, J. 2016. A user-developed, user run recovery programme for people with severe mental illness: A randomised control trial. *Psychosis* 2016;8(4):287-300 | Intervention does not include vocational component |
| 7 | Bowie, Christopher R.; Grossman, Michael; Gupta, Maya; Holshausen, Katherine; Best, Michael W. 2017. Action-Based Cognitive Remediation for Individuals With Serious Mental Illnesses: Effects of Real-World Simulations and Goal Setting on Functional and Vocational Outcomes. Psychiatric Rehabilitation Journal 2017;40(1):53-60 | Not randomised controlled trial |
| 8 | Burke-Miller, J.; Razzano, L.A.; Grey, D.D.; Blyler, C.R.; Cook, J.A. 2012. Supported employment outcomes for transition age youth and young adults *Psychiatric Rehabilitation Journal* 2012;35(3):171-179 | Not primary outcomes |
| 9 | Burns, Tom; Yeeles, Ksenija; Langford, Oliver; Montes, Maria Vazquez; Burgess, Jennifer; Anderson, Catriona. 2015 A randomised controlled trial of time-limited individual placement and support: IPS-LITE trial. *Br. J. Psychiatry* 2015;207(4):351-356 | Not people with autism, intellectual disability and/or psychosocial disability |
| 10 | Carlier, Bouwine E.; Schuring, Merel; Burdorf, Alex. 2018.Influence of an Interdisciplinary Re-employment Programme Among Unemployed Persons with Mental Health Problems on Health, Social Participation and Paid Employment. *Journal of Occupational Rehabilitation* 2018;28(1):147-157 | Not people with autism, intellectual disability and/or psychosocial disability |
| 11 | Chang, W.C.; Kwong, V.W.Y.; Or Chi Fai, P.; Lau, E.S.K.; Chan, G.H.K.; Jim, O.T.T.; Hui, C.L.M.; Chan, S.K.W.; Lee, E.H.M.; Chen, E.Y.H. 2018. Motivational impairment predicts functional | Not primary outcomes |

| | | |
|---|---|---|
| | remission in first-episode psychosis: 3-Year follow-up of the randomized controlled trial on extended early intervention. *Australian and New Zealand Journal of Psychiatry* 2018;52(12):1194-1201 | |
| 12 | Christensen, T.N.; Wallstrøm, I.G.; Stenager, E.; Bojesen, A.B.; Gluud, C.; Nordentoft, M.; Eplov, L.F. 2019. Effects of Individual Placement and Support Supplemented with Cognitive Remediation and Work-Focused Social Skills Training for People with Severe Mental Illness: A Randomized Clinical Trial. *JAMA Psychiatry* 2019;76(12):1232-1240 | Incorrect follow-up period |
| 13 | Chuang, Wen-Fang; Hwang, Eric; Lee, Hui-Ling; Wu, Shang-Liang. 2015. An In-House Prevocational Training Program for Newly Discharged Psychiatric Inpatients: Exploring Its Employment Outcomes and the Predictive Factors. *Occupational Therapy International* 2015;22(2):94-103 | No passive control or active control that fits intervention criteria |
| 14 | Cimera, Robert Evert; Wehman, Paul; West, Michael; Burgess, Sloane. 2012. Do sheltered workshops enhance employment outcomes for adults with autism spectrum disorder? *Autism* 2012;16(1):87-94 | Not randomised controlled trial |
| 15 | Contreras, Natalia A.; Castle, David J.; Crosse, Caroline; Morgain, Dea; Fossey, Ellie; Harvey, Carol; Rossell, Susan L. 2018. How Effective is Cognitive Remediation in Enhancing Vocational Outcomes for Job Seekers with Severe Mental Illness in Australia? *Australian Psychologist* 2018;53(2):144-150 | No passive control or active control that fits intervention criteria |
| 16 | Cook, Judith A.; Burke-Miller, Jane K.; Roessel, Emily. 2016. Long-Term Effects of Evidence-Based Supported Employment on Earnings and on SSI and SSDI Participation Among Individuals With Psychiatric Disabilities. *American Journal of Psychiatry* 2016;173(10):1007-1014 | Not people with autism, intellectual disability and/or psychosocial disability |
| 17 | Davis, L.L.; Resnick, S.G.; Maieritsch, K.P.; Weber, K.C.; Erbes, C.R.; Strom, T.Q.; McCall, K.P.; Kyriakides, T.C. 2019. Employment outcomes from VA vocational services involving transitional work for veterans with a diagnosis of posttraumatic stress disorder. *Psychiatric rehabilitation journal* 2019;42(3):257-267 | Not people with autism, intellectual disability and/or psychosocial disability |
| 18 | Davis, Lori L.; Kyriakides, Tassos C.; Suris, Alina M.; Ottomanelli, Lisa A.; Mueller, Lisa; Parker, Pamela E.; Resnick, Sandra G.; Toscano, Richard; Scrymgeour, Alexandra A.; Drake, Robert E. 2018. Effect of Evidence-Based Supported Employment vs Transitional Work on Achieving Steady Work Among Veterans With Posttraumatic Stress Disorder: A Randomized Clinical Trial. *JAMA Psychiatry* 2018;75(4):316-324 | Not people with autism, intellectual disability and/or psychosocial disability |
| 19 | Drake, Robert E.; Frey, William; Bond, Gary R.; Goldman, Howard H.; Salkever, David; Miller, Alexander; Moore, Troy A.; Riley, Jarnee; Karakus, Mustafa; Milfort, Roline. 2013. Assisting Social Security Disability Insurance Beneficiaries With Schizophrenia, Bipolar Disorder, or Major Depression in Returning to Work. *Am. J. Psychiat.* 2013;170(12):1433-1441 | Medical, pharmacological and/or psychological interventions are part of comparator or control condition only |

| | | |
|---|---|---|
| 20 | Eack, S.M.; Hogarty, G.E.; Greenwald, D.P.; Hogarty, S.S.; Keshavan, M.S. 2011. Effects of cognitive enhancement therapy on employment outcomes in early schizophrenia: Results from a 2-year randomized trial. *Research on Social Work Practice* 2011;21(1):32-42 | Medical, pharmacological and/or psychological interventions are part of comparator or control condition only |
| 21 | Eack, S.M.; Hogarty, S.S.; Greenwald, D.P.; Litschge, M.Y.; Porton, S.A.; Mazefsky, C.A.; Minshew, N.J. 2018. Cognitive enhancement therapy for adult autism spectrum disorder: Results of an 18-month randomized clinical trial. *Autism Research* 2018;11(3):519-530 | Intervention does not include vocational component |
| 22 | Eack, Shaun M; Hogarty, Gerard E; Greenwald, Deborah P; Hogarty, Susan S; Keshavan, Matcheri S. 2011. Effects of Cognitive Enhancement Therapy on Employment Outcomes in Early Schizophrenia: Results From a Two-Year Randomized Trial. *Res Soc Work Pract* 2011;21(1):32-42 | Intervention does not include vocational component |
| 23 | Economou, Marina; Palli, Alexandra; Peppou, Lily; Madianos, Michael. 2011. Recovery from schizophrenia: A four-year study of an inner city cohort. *Community Mental Health Journal* 2011;47(Health & Mental Health Treatment & Prevention [3300]):660-667 | No passive control or active control that fits intervention criteria |
| 24 | Ellison, Marsha Langer; Rogers, E. Sally; Lyass, Asya; Massaro, Joseph; Wewiorski, Nancy J.; Hsu, Su-Ting; Anthony, William A. 2011. Statewide Initiative of Intensive Psychiatric Rehabilitation: Outcomes and Relationship to Other Mental Health Service Use. *Psychiatric Rehabilitation Journal* 2011;35(1):9-19 | Intervention does not include vocational component |
| 25 | Falkum, E.; Klungsøyr, O.; Lystad, J.U.; Bull, H.C.; Evensen, S.; Martinsen, E.W.; Friis, S.; Ueland, T. 2017. Vocational rehabilitation for adults with psychotic disorders in a Scandinavian welfare society. *BMC Psychiatry* 2017;17(1): | Not randomised controlled trial |
| 26 | Ferguson, Kristin M. 2018. Employment Outcomes From a Randomized Controlled Trial of Two Employment Interventions With Homeless Youth. *J. Soc. Soc. Work Res*. 2018;9(1):1-21 | Not primary outcomes |
| 27 | Ferguson, Kristin; Xie, Bin; Glynn, Shirley. 2012. Adapting the Individual Placement and Support Model with Homeless Young Adults. *Child & Youth Care Forum* 2012;41(3):277-294 | Not randomised controlled trial |
| 28 | Fyhn, T.; Øverland, S.; Reme, S.E. 2020. Predictors of employment in people with moderate to severe mental illness participating in a randomized controlled trial of Individual Placement and Support (IPS). *International Journal of Social Psychiatry* 2020;(): | Not primary outcomes |
| 29 | Germundsson, Per; Gustafsson, Johanna; Lind, Martin; Danermark, Berth. 2012. Disability and supported employment: impact on employment, income, and allowances. *International Journal of Rehabilitation Research* 2012;35(3):263-269 | Not randomised controlled trial |
| 30 | Glynn, S.M.; Marder, S.R.; Noordsy, D.L.; O'Keefe, C.; Becker, D.R.; Drake, R.E.; Sugar, C.A. 2017. An RCT evaluating the effects of skills training and medication type on work outcomes among patients with schizophrenia. *Psychiatric Services* 2017;68(3):271-277 | Medical, pharmacological and/or psychological interventions are part of comparator or control condition only |
| 31 | Gorenstein, M.; Giserman-Kiss, I.; Feldman, E.; Isenstein, E.L.; Donnelly, L.; Wang, A.T.; Foss-Feig, J.H. 2020. Brief Report: A Job-Based Social Skills Program (JOBSS) for Adults with Autism Spectrum Disorder: A Pilot Randomized Controlled Trial. *Journal of Autism and Developmental Disorders* 2020;(): | Not randomised controlled trial |

| | | |
|---|---|---|
| 32 | Gray, Heather M; Nelson, Sarah E; Shaffer, Howard J; Stebbins, Patricia; Farina, Andrea Ryan. 2017. How do homeless adults change their lives after completing an intensive job-skills program? A prospective study. *Journal of Community Psychology* 2017;45(7):888-905 | Not randomised controlled trial |
| 33 | Hampson, Margaret E.; Hicks, Richard E.; Watt, Bruce D. 2015. Exploring the Effectiveness of Motivational Interviewing in Re-engaging People Diagnosed with Severe Psychiatric Conditions in Work, Study, or Community Participation. *American Journal of Psychiatric Rehabilitation* 2015;18(3):265-279 | Not randomised controlled trial |
| 34 | Hara, Karen Walseth; Bjørngaard, Johan Håkon; Jacobsen, Henrik Børsting; Borchgrevink, Petter C.; Johnsen, Roar; Stiles, Tore C.; Brage, Søren; Woodhouse, Astrid. 2018. Biopsychosocial predictors and trajectories of work participation after transdiagnostic occupational rehabilitation of participants with mental and somatic disorders: a cohort study. *BMC Public Health* 2018;18(1):N.PAG-N.PAG | Not people with autism, intellectual disability and/or psychosocial disability |
| 35 | Harris, Anthony W. F.; Kosic, Tanya; Xu, Jean; Walker, Chris; Gye, William; Hodge, Antoinette Redoblado. 2017. Web-Based Cognitive Remediation Improves Supported Employment Outcomes in Severe Mental Illness: Randomized Controlled Trial. *JMIR Ment. Health* 2017;4(3):UNSP-e30 | Intervention does not include vocational component |
| 36 | Hees, H.L.; De Vries, G.; Koeter, M.W.J.; Schene, A.H. 2013. Adjuvant occupational therapy improves long-term depression recovery and return-to-work in good health in sick-listed employees with major depression: Results of a randomised controlled trial. *Occupational and Environmental Medicine* 2013;70(4):252-260. | Not primary outcomes |
| 37 | Hellerstein, D.J.; Erickson, G.; Stewart, J.W.; McGrath, P.J.; Hunnicutt-Ferguson, K.; Reynolds, S.K.; O'Shea, D.; Chen, Y.; Withers, A.; Wang, Y. 2015. Behavioral activation therapy for return to work in medication-responsive chronic depression with persistent psychosocial dysfunction. *Comprehensive Psychiatry* 2015;57():140-147 | Not randomised controlled trial |
| 38 | Hellström, L.; Bech, P.; Hjorthøj, C.; Nordentoft, M.; Lindschou, J.; Eplov, L.F. 2017. Effect on return to work or education of individual placement and support modified for people with mood and anxiety disorders: Results of a randomised clinical trial. *Occupational and Environmental Medicine* 2017;74(10):717-725 | Not people with autism, intellectual disability and/or psychosocial disability |
| 39 | Humensky, Jennifer L.; Essock, Susan M.; Dixon, Lisa B. 2017. Characteristics Associated With the Pursuit of Work and School Among Participants in a Treatment Program for First Episode of Psychosis. *Psychiatric Rehabilitation Journal* 2017;40(1):108-112 | No passive control or active control that fits intervention criteria |
| 40 | Kane, John M.; Robinson, Delbert G.; Schooler, Nina R.; Mueser, Kim T.; Penn, David L.; Rosenheck, Robert A.; Addington, Jean; Brunette, Mary F.; Correll, Christoph U.; Estroff, Sue E.; Marcy, Patricia; Robinson, James; Meyer-Kalos, Piper S.; Gottlieb, Jennifer D.; Glynn, Shirley M.; Lynde, David W.; Pipes, Ronny; Kurian, Benji T.; Miller, Alexander L.; Azrin, Susan T. 2016. | Medical, pharmacological and/or psychological interventions are part of comparator or control condition only |

| | | |
|---|---|---|
| | Comprehensive Versus Usual Community Care for First-Episode Psychosis: 2-Year Outcomes From the NIMH RAISE Early Treatment Program. *American Journal of Psychiatry* 2016;173(4):362-372 | |
| 41 | Khalifa, N.; Talbot, E.; Barber, S.; Schneider, J.; Bird, Y.; Attfield, J.; Bates, P.; Walker, D.-M.; Völlm, B. 2020. A Feasibility Cluster Randomized Controlled Trial of Individual Placement and Support (IPS) for Patients With Offending Histories. *Frontiers in Psychiatry* 2020;10(): | Not people with autism, intellectual disability and/or psychosocial disability |
| 42 | Kilian, R.; Lauber, C.; Kalkan, R.; Dorn, W.; Rössler, W.; Wiersma, D.; Van Buschbach, J.T.; Fioritti, A.; Tomov, T.; Catty, J.; Burns, T.; Becker, T. 2012. The relationships between employment, clinical status, and psychiatric hospitalisation in patients with schizophrenia receiving either IPS or a conventional vocational rehabilitation programme. *Social Psychiatry and Psychiatric Epidemiology* 2012;47(9):1381-1389 | Not primary outcomes |
| 43 | Knapp, M.; Patel, A.; Curran, C.; Latimer, E.; Catty, J.; Becker, T.; Drake, R.E.; Fioritti, A.; Kilian, R.; Lauber, C.; RöSSLER, W.; Tomov, T.; Van Busschbach, J.; Comas-Herrera, A.; White, S.; Wiersma, D.; Burns, T. 2013. Supported employment: Cost-effectiveness across six European sites. *World Psychiatry* 2013;12(1):60-68 | Not primary outcomes |
| 44 | Kukla, M.; Bell, M.D.; Lysaker, P.H. 2018. A randomized controlled trial examining a cognitive behavioral therapy intervention enhanced with cognitive remediation to improve work and neurocognition outcomes among persons with schizophrenia spectrum disorders. *Schizophrenia Research* 2018;197():400-406 | Not primary outcomes |
| 45 | Kukla, Marina; Salyers, Michelle P.; Strasburger, Amy M.; Johnson-Kwochka, Annalee; Amador, Emily; Lysaker, Paul H. 2019. Work-Focused Cognitive Behavioral Therapy to Complement Vocational Services for People With Mental Illness: Pilot Study Outcomes Across a 6-Month Posttreatment Follow-Up. *Psychiatric Rehabilitation Journal* 2019;42(4):366-371 | No passive control or active control that fits intervention criteria |
| 46 | Kukla, Marina; Strasburger, Amy M.; Salyers, Michelle P.; Rollins, Angela L.; Lysaker, Paul H. 2018. A Pilot Test of Group Based Cognitive Behavioral Therapy to Augment Vocational Services for Persons With Serious Mental Illness: Feasibility and Competitive Work Outcomes. *Journal of Nervous & Mental Disease* 2018;206(5):310-315 | No passive control or active control that fits intervention criteria |
| 47 | Lammerts, Lieke; Schaafsma, Frederieke G; Bonefaas-Groenewoud, Karin; van Mechelen, Willem; Anema, Johannes. 2016. Effectiveness of a return-to-work program for workers without an employment contract, sick-listed due to common mental disorders. *Scand J Work Environ Health* 2016;42(6):469-480 | Not people with autism, intellectual disability and/or psychosocial disability |
| 48 | Lammerts, Lieke; van Dongen, Johanna M.; Schaafsma, Frederieke G.; van Mechelen, Willem; Anema, Johannes R. 2017. A participatory supportive return to work program for workers without an employment contract, sick-listed due to a common mental disorder: an economic evaluation alongside a randomized controlled trial. *BMC Public Health* 2017;17(1):1-13 | Not people with autism, intellectual disability and/or psychosocial disability |

| | | |
|---|---|---|
| 49 | Major, Barnaby S; Hinton, Mark F; Flint, Amy; Chalmers-Brown, Anna; McLoughlin, Katharine; Johnson, Sonia. 2010. Evidence of the effectiveness of a specialist vocational intervention following first episode psychosis: A naturalistic prospective cohort study. *Social Psychiatry and Psychiatric Epidemiology*: 2010;45(1):1-8 | Not randomised controlled trial |
| 50 | Martin, Marie; Nielsen, Maj; Madsen, Ida; Petersen, Signe; Lange, Theis; Rugulies, Reiner. 2013.Effectiveness of a Coordinated and Tailored Return-to-Work Intervention for Sickness Absence Beneficiaries with Mental Health Problems. *Journal of Occupational Rehabilitation* 2013;23(4):621-630 | Not randomised controlled trial |
| 51 | McFarlane, William R.; Levin, Bruce; Travis, Lori; Lucas, F. Lee; Lynch, Sarah; Verdi, Mary; Williams, Deanna; Adelsheim, Steven; Calkins, Roderick; Carter, Cameron S.; Cornblatt, Barbara; Taylor, Stephan F.; Auther, Andrea M.; McFarland, Bentson; Melton, Ryan; Migliorati, Margaret; Niendam, Tara; Ragland, J. Daniel; Sale, Tamara; Salvador, Melina; Spring, Elizabeth. 2015. Clinical and Functional Outcomes After 2 Years in the Early Detection and Intervention for the Prevention of Psychosis Multisite Effectiveness Trial. *Schizophr. Bull.* 2015;41(1):30-43 | Not people with autism, intellectual disability and/or psychosocial disability |
| 52 | McGurk, S.R.; Mueser, K.T.; Xie, H.; Feldman, K.; Shaya, Y.; Klein, L.; Wolfe, R. 2016. Cognitive remediation for vocational rehabilitation nonresponders. *Schizophrenia Research* 2016;175(1-3):48-56 | Incorrect follow-up period |
| 53 | McGurk, Susan R; Mueser, Kim T; Watkins, Melanie A; Dalton, Carline M; Deutsch, Heather. 2017. The feasibility of implementing cognitive remediation for work in community based psychiatric rehabilitation programs. *Special Issue: Cognitive Remediation* 2017;40(1):79-86 | Not randomised controlled trial |
| 54 | Mueser, K.T.; Bond, G.R.; Essock, S.M.; Clark, R.E.; Carpenter-Song, E.; Drake, R.E.; Wolfe, R. 2014. The effects of supported employment in Latino consumers with severe mental illness. *Psychiatric Rehabilitation Journal* 2014;37(2):113-122 | Not randomised controlled trial |
| 55 | Nossel, Ilana; Wall, Melanie M.; Scodes, Jennifer; Marino, Leslie A.; Zilkha, Sacha; Bello, Iruma; Malinovsky, Igor; Lee, Rufina; Radigan, Marleen; Smith, Thomas E.; Sederer, Lloyd; Gu, Gyojeong; Dixon, Lisa. 2018. Results of a Coordinated Specialty Care Program for Early Psychosis and Predictors of Outcomes. *Psychiatric Services* 2018;69(8):863-870 | No passive control or active control that fits intervention criteria |
| 56 | Overland, Simon; Grasdal, Astrid Louise; Reme, Silje Endresen. 2018. Long-term effects on income and sickness benefits after work-focused cognitive-behavioural therapy and individual job support: a pragmatic, multicentre, randomised controlled trial. *Occup Environ Med* 2018;75(10):703-708 | Not people with autism, intellectual disability and/or psychosocial disability |
| 57 | Parletta, Vanessa A; Waghorn, Geoffrey; Dias, Shannon. 2017. The applicability of supported employment to adults with participation obligations as a condition for receiving welfare benefits. *American Journal of Psychiatric Rehabilitation* 2017;20(2):106-125 | Not randomised controlled trial |
| 58 | Poremski, Daniel; Stergiopoulos, Vicky; Braithwaite, Erika; Distasio, Jino; Nisenbaum, Rosane; Latimer, Eric. 2016. Effects of Housing First on Employment and Income of Homeless Individuals: Results of a Randomized Trial. *Psychiatr. Serv*. 2016;67(6):602-608 | Intervention does not include vocational component |

| | | |
|---|---|---|
| 59 | Reme, S.E.; Monstad, K.; Fyhn, T.; Sveinsdottir, V.; Løvvik, C.; Lie, S.A.; Øverland, S. 2019. A randomized controlled multicenter trial of individual placement and support for patients with moderate-to-severe mental illness. *Scandinavian Journal of Work, Environment and Health* 2019;45(1):33-41 | Not people with autism, intellectual disability and/or psychosocial disability |
| 60 | Reme, Silje Endresen; Grasdal, Astrid Louise; Løvvik, Camilla; Lie, Stein Atle; Øverland, Simon. 2015. Work-focused cognitive-behavioural therapy and individual job support to increase work participation in common mental disorders: a randomised controlled multicentre trial. *Occupational & Environmental Medicine* 2015;72(10):745-752 | Not people with autism, intellectual disability and/or psychosocial disability |
| 61 | Rodríguez Pulido, F.; Caballero Estebaranz, N.; González Dávila, E.; Melián Cartaya, M.J. 2019. Cognitive remediation to improve the vocational outcomes of people with severe mental illness. *Neuropsychological Rehabilitation* 2019;(): | Intervention does not include vocational component |
| 62 | Rodríguez Pulido, F.; Caballero Estebaranz, N.; Tallo Aldana, E.; Méndez Abad, M.E.; Hernández Álvarez-Sotomayor, M.C.; López Reig, S.; Vílchez de León, P.I.; González-Dávila, E. 2018. Effectiveness of individual supported employment for people with severe mental disorder. *Gaceta Sanitaria* 2018;32(6):513-518 | Not in English |
| 63 | Roessler, Wulf; Kawohl, Wolfram; Nordt, Carlos; Haker, Helene; Ruesch, Nicolas; Hengartner, Michael P. 2018. "Placement Budgets" for Supported Employment-Impact on Quality of Life in a Multicenter Randomized Controlled Trial. *Front. Psychiatry* 2018;9():462 | Not primary outcomes |
| 64 | Rosen, M.I.; Ablondi, K.; Black, A.C.; Mueller, L.; Serowik, K.L.; Martino, S.; Mobo, B.H.; Rosenheck, R.A. 2014. Work outcomes after benefits counseling among veterans applying for service connection for a psychiatric condition. *Psychiatric Services* 2014;65(12):1426-1432 | Not people with autism, intellectual disability and/or psychosocial disability |
| 65 | Rosenheck, R.; Mueser, K.T.; Sint, K.; Lin, H.; Lynde, D.W.; Glynn, S.M.; Robinson, D.G.; Schooler, N.R.; Marcy, P.; Mohamed, S.; Kane, J.M. 2017. Supported employment and education in comprehensive, integrated care for first episode psychosis: Effects on work, school, and disability income. *Schizophrenia Research* 2017;182():120-128 | Medical, pharmacological and/or psychological interventions are part of comparator or control condition only |
| 66 | Rosenheck, Robert A.; Estroff, Sue E.; Sint, Kyaw; Lin, Haiqun; Mueser, Kim T.; Robinson, Delbert G.; Schooler, Nina R.; Marcy, Patricia; Kane, John M. 2017. Incomes and Outcomes: Social Security Disability Benefits in First-Episode Psychosis. *Am. J. Psychiat*. 2017;174(9):886-894 | Not primary outcomes |
| 67 | Rossler, Wulf; Kawohl, Wolfram; Nordt, Carlos; Haker, Helene; Rusch, Nicolas; Hengartner, Michael P. 2020. 'Placement budgets' for supported employment: impact on employment rates in a multicentre randomised controlled trial. *Br. J. Psychiatry* 2020;216(6):308-313 | Not people with autism, intellectual disability and/or psychosocial disability |
| 68 | Sanches, S.A.; Swildens, W.E.; Schaefer, B.; Moerbeek, M.; Feenstra, T.L.; van Asselt, A.D.I.; Danner, U.N.; van Weeghel, J.; van Busschbach, J.T. 2020. Effectiveness of the Boston University Approach to Psychiatric Rehabilitation in Improving Social Participation in People With Severe Mental Illnesses: A Randomized Controlled Trial. *Frontiers in Psychiatry* 2020;11(): | Not primary outcomes |

| | | |
|---|---|---|
| 69 | Sánchez, Jennifer. 2018. Employment predictors and outcomes of U.S. state-federal vocational rehabilitation consumers with affective disorders: A CHAID analysis. *Journal of Affective Disorders* 2018;239():48-57 | Not randomised controlled trial |
| 70 | Schall, C.; Sima, A.P.; Avellone, L.; Wehman, P.; McDonough, J.; Brown, A. 2020. The effect of business internships model and employment on enhancing the independence of young adults with significant impact from autism. *Intellectual and Developmental Disabilities* 2020;58(4):301-313 | Not primary outcomes |
| 71 | Smith, M.J.; Fleming, M.F.; Wright, M.A.; Losh, M.; Humm, L.B.; Olsen, D.; Bell, M.D. 2015. Brief Report: Vocational Outcomes for Young Adults with Autism Spectrum Disorders at Six Months After Virtual Reality Job Interview Training. *Journal of Autism and Developmental Disorders* 2015;45(10):3364-3369 | Not primary outcomes |
| 72 | Smith, M.J.; Smith, J.D.; Fleming, M.F.; Jordan, N.; Oulvey, E.A.; Bell, M.D.; Mueser, K.T.; McGurk, S.R.; Spencer, E.-S.; Mailey, K.; Razzano, L.A. 2019. Enhancing individual placement and support (IPS) - Supported employment: A Type 1 hybrid design randomized controlled trial to evaluate virtual reality job interview training among adults with severe mental illness. *Contemporary Clinical Trials* 2019;77():86-97 | Not randomised controlled trial |
| 73 | Srihari, V.H.; Tek, C.; Kucukgoncu, S.; Phutane, V.H.; Breitborde, N.J.K.; Pollard, J.; Ozkan, B.; Saksa, J.; Walsh, B.C.; Woods, S.W. 2015. First-episode services for psychotic disorders in the U.S. public sector: A pragmatic randomized controlled trial. *Psychiatric Services* 2015;66(7):705-712 | Medical, pharmacological and/or psychological interventions are part of comparator or control condition only |
| 74 | Sutton, Rebecca; Lawrence, Kate; Zabel, Elisabeth; French, Paul. 2019. Recovery College influences upon service users: a Recovery Academy exploration of employment and service use. *Journal of Mental Health Training, Education & Practice* 2019;14(3):141-148 | Not people with autism, intellectual disability and/or psychosocial disability |
| 75 | Sveinsdottir, V.; Lie, S.A.; Bond, G.R.; Eriksen, H.R.; Tveito, T.H.; Grasdal, A.L.; Reme, S.E. 2020. Individual placement and support for young adults at risk of early work disability (The SEED trial). a randomized controlled trial. *Scandinavian Journal of Work, Environment and Health* 2020;46(1):50-59 | Not people with autism, intellectual disability and/or psychosocial disability |
| 76 | Swildens, Wilma; van Busschbach, Jooske T.; Michon, Harry; Kroon, Hans; Koeter, Maarten W. J.; Wiersma, Durk; van Os, Jim. 2011. Effectively Working on Rehabilitation Goals: 24-Month Outcome of a Randomized Controlled Trial of the Boston Psychiatric Rehabilitation Approach. *Can. J. Psychiat.-Rev. Can. Psychiat*. 2011;56(12):751-760 | Not primary outcomes |
| 77 | Tan, B.-L.; King, R. 2013. The effects of cognitive remediation on functional outcomes among people with schizophrenia: A randomised controlled study. *Australian and New Zealand Journal of Psychiatry* 2013;47(11):1068-1080 | Intervention does not include vocational component |
| 78 | Tsang H.W.; Bell M.D.; Cheung V.; Tam K.L.; Yeung W.S. 2016. Integrated supported employment plus cognitive remediation training for people with schizophrenia. *Hong Kong Med J* 2016;22(Supplement 2):S15-S18 | Intervention does not include vocational component |

| | | |
|---|---|---|
| 79 | Tsang, H W H. 2011. Supported employment versus traditional vocational rehabilitation for individuals with severe mental illness: a three-year study. *HONG KONG MED. J*. 2011;17 Suppl 2(dnz, 9512509):13-7 | Not primary outcomes |
| 80 | Tsang, H W H; Bell, M D; Cheung, V; Tam, K L; Yeung, W S. 2016. Integrated supported employment plus cognitive remediation training for people with schizophrenia. *HONG KONG MED. J.* 2016;22 Suppl 2(dnz, 9512509):S15-8 | Intervention does not include vocational component |
| 81 | Turner, Niall; Nesbitt, Tara; Fanning, Felicity; Clarke, Mary. 2019. Improving vocational recovery among people with psychosis: a two-pronged approach. *Irish Journal of Occupational Therapy* 2019;47(2):114-123 | No passive control or active control that fits intervention criteria |
| 82 | Twamley, E.W.; Thomas, K.R.; Burton, C.Z.; Vella, L.; Jeste, D.V.; Heaton, R.K.; McGurk, S.R. 2019. Compensatory cognitive training for people with severe mental illnesses in supported employment: A randomized controlled trial. *Schizophrenia Research* 2019;203():41-48 | Medical, pharmacological and/or psychological interventions are part of comparator or control condition only |
| 83 | van Veggel, Rhonda; Waghorn, Geoffrey; Dias, Shannon. 2015. Implementing evidence-based supported employment in Sussex for people with severe mental illness. *British Journal of Occupational Therapy* 2015;78(5):286-294 | Not randomised controlled trial |
| 84 | Wells, Kenneth B; Jones, Loretta; Chung, Bowen; Dixon, Elizabeth L; Tang, Lingqi; Gilmore, Jim; Sherbourne, Cathy; Ngo, Victoria K; Ong, Michael K; Stockdale, Susan; Ramos, Esmeralda; Belin, Thomas R; Miranda, Jeanne. 2013. Community-partnered cluster-randomized comparative effectiveness trial of community engagement and planning or resources for services to address depression disparities. *JGIM: Journal of General Internal Medicine* 2013;28(10):1268-1278 | Not people with autism, intellectual disability and/or psychosocial disability |
| 85 | Young, Daniel K. W.; Ng, Petrus Y. N.; Cheng, Daphne; Leung, C. H. 2019. A Vocational Recovery Model for Young People With Mental Illness: A Pretest–Posttest. *Research on Social Work Practice* 2019;29(5):495-505 | Not randomised controlled trial |
| 86 | Young, K. W; Ng, P; Pan, J. 2014. Functional recovery of consumers discharged from mental hospital and participating in a community-based psychosocial programme provided by a non-governmental organisation. *East Asian Archives of Psychiatry* 2014;24(4):139-147 | Not randomised controlled trial |
