# Supplementary material for: Vocational Interventions to Improve Employment Participation of People with Psychosocial Disability, Autism and/or Intellectual Disability: A Systematic Review
Source: Int J Environ Res Public Health. 2021 Nov 17;18(22):12083. doi: 10.3390/ijerph182212083 (PMC8618542; doi:10.3390/ijerph182212083)
Supplement: Supplementary file 1 [file ijerph-18-12083-s001.zip › Supplementary File S5_interventions all studies_26_7_2021.pdf]

Supplementary File S5: Descriptions of Interventions in included studies

**Studies including people with psychosocial disabilities**		
**Study**	**Interventions**	**Control / Comparator**
Bejerholm 2015	**IPS (Good to excellent IPS fidelity)**<br>IPS is characterised by eight principles: (1) focus on open employment outcomes, (2) open to anyone who wants to work (3) client preferences guide decisions, (4) rapid job search, (5) systematic job development, (6) time-unlimited individualised supports, (7) integrated with mental health teams, and (8) benefits counselling.<br>Three employment specialists were recruited, with a caseload ratio of 20 participants per 1 fulltime employment specialist.	**Traditional vocational rehabilitation**<br>'Train-place' vocational services, which commonly involve prevocational training in sheltered settings in a stepwise manner. Services ranged from individual rehabilitation support from a team member in the mental healthcare service, municipality-run sheltered or day centre activities and prevocational training, joint co-operation of vocational service in the Social Insurance Agency/ Public Employment Service, and support from either the Public Employment Service or the Social Insurance Agency. Some participants enrolled themselves in Fountain House (clubhouse) activities. None of the services were delivering IPS according to the Supported Employment Fidelity Scale.
Bond 2015	**IPS (Good IPS fidelity)**<br>IPS was delivered by a team of full-time employment specialists, each with a caseload of 20 participants. The employment specialists received a day-long training on criminal justice issues. IPS involved brief vocational assessment, followed by rapid job search based on participant preferences, and job development by employment specialist who offered to accompany participants to interviews. Employment specialists were the main source of support for participants.	**Work Choice (Adequate Work Choice fidelity)**<br>Work Choice is based on the job club model and tailored for people with psychiatric disabilities. It is characterised by a self-directed job search and assistance with résumé preparation, interview skills, and job leads. The curriculum included training in application procedures, job search strategies, and linkage services. Classes were scheduled weekly at two conveniently located sites. Two half-time workers staffed the program, with a caseload ratio of 40 clients per 1.0 FTE. Peer support was the main source of support for participants.
Craig 2014	**IPS and motivational interviewing (Good to exemplary IPS fidelity)**<br>Care coordinators received motivational interviewing training from recognised experts. The training aimed to provide clinicians with a clear understanding of issues such as intrinsic motivation, ambivalence and readiness to change, as well as how to influence conversations, recognise appropriate times to use motivational interviewing and feel confident in the use of motivational interviewing in everyday practice.	**IPS only (Good to exemplary IPS fidelity)**<br>The employment specialists were trained to deliver IPS.
Davis 2012	**IPS (Fair IPS fidelity)**<br>The principles of IPS were followed.	**Veteran Affairs Vocational Rehabilitation Program**<br>This variable time-limited service included at least one of the following: (1) routine prevocational testing and evaluation for all referred participants, (2) vocational rehabilitation therapy that provided a work regimen with monetary incentives derived from contracts whereby participants are paid on a piece-rate basis related to their production, and (3) a transitional work program that included a temporary work experience either within the Tuscaloosa Veteran Affairs Medical Centre or in community settings and was not necessarily individualised to the participants' preferences. This program had very little integration with the mental health treatment team. The Vocational Rehabilitation Program specialist provided support during the set-aside work experience and provided job search assistance once the work experience

		neared completion. There was little or no vocational assistance after open employment was obtained. The specialist had no maximum caseload. The Vocational Rehabilitation Program was consistently rated by the IPS fidelity monitor as ≤40 during the study, which is appropriate for this intervention.
Davis 2018	**Vocational Coach**<br>Multisystemic therapy for emerging adults vocational coaches delivered the standard skills curricula enhanced with vocational components based on participants' desired outcomes, treatment plan and needs. Vocational domains of the curricula were prioritised above other domains. Participants were not connected to state vocational rehabilitation services	**Standard Coach plus vocational rehabilitation**<br>Multisystemic Therapy for emerging adults standard coaches delivered standard skills curricula based on participants' desired outcomes, treatment plan and needs. If participants were interested in employment, their coach referred them to state vocational rehabilitation services for typical vocational support.
Erickson 2020	**IPS,** good IPS fidelity<br>Participants received employment support from one of two experienced vocational counsellors. Both IPS workers provided services in a range of locations, both within and outside of the mental health centres.	**Treatment as usual**<br>No constraints on the use of other employment support services
Hoffmann 2012, 2014	**Job Coach Project** (good IPS fidelity)<br>The Job Coach Project of the Bern University Hospital of Psychiatry was derived from the individual placement and support model of supported employment. Modifications were made in order to improve sustainability, and meet the standards of the Swiss social insurance system and the needs of the Swiss labour market. Some incentives were given to employers of participants in the Job Coach Project. The Job Coach Project was staffed by employment specialists who had a maximum caseload of 15 participants. Participants were assisted by an employment specialist in a rapid job search based on their educational background, work preference, and previous work experience. Once employed, on-the-job training and support is provided to facilitate job maintenance. In the event of job loss, support and assistance are provided to obtain employment. Employment specialists had contact at least once a fortnight with participants.	**Traditional vocational rehabilitation**<br>Traditional vocational rehabilitation programs in Switzerland are based on a "train-place" approach. Each program participating in this study was deemed by the Federal Social Insurance Office to be the best locally available alternative for the prospective participant. All participants received prevocational training in sheltered workshops for 6 to 12 months. If feasible, participants then completed 3 to 6 months of training in an open market job. Support ceased at the end of the traditional rehabilitation program regardless of whether the participant obtained open employment.
Howard 2010, Heslin 2011	**IPS, high IPS fidelity**<br>IPS programme integrated within community mental health teams provided by a well-established not-for-profit non-governmental supported employment agency. The intervention involved linking four experienced employment specialists (two for each borough) with community mental health teams. The employment focused on rapid placement with continued follow-up support and sought to find employment opportunities that were consistent with participants' preferences, skills and abilities. However, the integration of the IPS programme was not structural or managerial, reflecting supported employment as it is provided in the vast majority of settings in the UK, which is the current provision referred to by the UK government in its implementation of IPS.	**Traditional vocational rehabilitation**<br>Local traditional vocational services consisted of existing psychosocial rehabilitation and day care programmes available in the local area. These services most commonly offered pre-employment preparation (e.g. interview skills, curriculum vitae coaching and application form practise), computers/information technology and confidence building/motivation.
Killackey 2019	**IPS (Good IPS fidelity)**<br>IPS was delivered by a vocational specialist who had a background working in general and disability employment. In keeping with the IPS principles the vocational specialist was embedded as a member of the clinical team.	**Referral to external government-contracted employment agencies**<br>Treatment as usual in Australia involves referral to external government-contracted employment agencies. There is typically little follow-up between mental health and employment agencies. The burden of navigating the different systems often falls on the individual. In Australia, people with a mental illness are able to opt out of any welfare-

		related obligation to seek employment via certification of a medical condition from their medical practitioner.<br><br>**Note:** Participants in both conditions continued to receive standard Early Psychosis Prevention and Intervention Centre treatment, including medical management and review, out-patient case management, access to Early Psychosis Prevention and Intervention Centre group programme and peer and family support.
Lecomte 2020	**Cognitive behaviour therapy group intervention adapted for supported employment programs plus supported employment program**<br>Participants received a maximum of eight 1-hour group sessions, twice per week for one month. Participants had their own manual that contained information on all eight sessions, as well as homework to be completed outside of the sessions. Each session was led by two co-therapists and included an average of five participants per group. Sessions usually involved review of the homework, presentation of the theme of the session, presentation of some didactic information, discussions and application to self of the material, writing relevant information to retain into one's manual, review of the session and presentation of the homework. The last session closes with a graduation celebration.	**Supported employment program only**<br>Note: no further description was given in article
McGurk 2015	**Enhanced supported employment plus the Thinking Skills for Work Program**<br>The Thinking Skills for Work program uses three strategies to increase cognitive performance at work: (1) cognitive exercise practice, (2) strategy coaching, and (3) teaching coping/compensatory strategies. The cognitive specialist had contact with participants in the Thinking Skills for Work Program and served as a member of the employment team. This involved updating the team about participants' progress, learning about their difficulties with employment, recommending compensatory strategies for reducing the impact of cognitive challenges, and problem solving related to implementing these and other strategies.	**Enhanced supported employment only**<br>Supported employment followed the individual placement and support model. Supported employment was enhanced by training employment specialists on cognitive impairments that interfere with work performance and strategies to help participants cope. Employment specialists had caseloads of 20 participants. Each employment specialist served participants in both treatment conditions. Potential for contamination between conditions was minimised by providing the manual for the Thinking Skills for Work program only to the cognitive specialist who implemented the program.
Michon 2014	**IPS (moderate to good fidelity)**<br>IPS was implemented according to protocol. Employment specialists assisted people in getting regular jobs, offered follow-along support, spent most of the time in the community and operated in close collaboration with the other community mental health team members. Employment specialists were placed in community mental health teams with a staff: client ratio ranging from 1:20 to 1:30. The majority of mental health services and treatment offered by these outpatient teams were provided in the community, employing assertive outreach. Four IPS services were trained and monitored on model.	**Traditional vocational rehabilitation**<br>Traditional vocational rehabilitation was facilitated by the mental health agency in a separate rehabilitation centre or by public services aimed at vocational rehabilitation. These services offer a stepwise vocational trajectory, putting much stronger emphasis on lengthy assessment of individual competencies and on connecting to pre- vocational activities such as voluntary jobs before placement in regular jobs. These program characteristics are in contrast with the rapid job search, very short assessment and minimum of prevocational training in IPS. Traditional vocational rehabilitation staff did not participate in the mental health teams. Traditional vocational rehabilitation can be described as a separated train-then-place model. Control services were assessed once with the Quality of Supported Employment Implementation Scale during the middle of the data collection phase. One of the centres with moderate IPS fidelity showed a minimal contrast with the control condition. The three other sites showed adequate fidelity contrasts in the two conditions.
Nuechterlein 2020	**IPS plus Workplace Fundamentals Module**, good IPS fidelity	**Conventional Brokered Vocational Rehabilitation plus social skills training intervention**

	The intervention followed the principles of IPS, but was adapted to provide supported education for participants whose preferences and situations made resuming education more appropriate than employment. The employment specialist was a member of the clinical team, and met with the participants. The Workplace Fundamentals Module used a group-based skills training approach with a focus on the social and problem-solving skills required for employment. Skill areas included: (1) how work/ school changes your life, (2) learning about your place of work or school, (3) identifying stressors, (4) solving problems, (5) managing symptoms and medications, (6) managing health, (7) improving job/school performance, (8) socialising with fellow workers/students, and (9) finding motivation. Each skill involved showing videotaped scenarios, role played practice, generation and evaluation of solutions to individually relevant school/work problems, and individualised homework. Additional scenarios were generated to adapt the Workplace Fundamentals Module to school settings. Participants used the Workplace Fundamentals Module Job Organizing Book during the group sessions, led by the case managers. Participants had 75 min groups once a week for 6 months, followed by booster groups of fading frequency over 6 months.	Case managers made referrals to vocational rehabilitation services at separate agencies. The vocational rehabilitation at local state agencies emphasised initial assessment of vocational abilities and interests, referrals to job openings and school opportunities, and arrangements to pay for schooling or job training, but not in-person, active outreach in the community. The study case managers ensured that participants had an initial appointment and served as the communications link with the vocational rehabilitation centre. Participants also participated in clinic-based skills training groups, matched in time to the Workplace Fundamentals Module. Skills training included medication management and communication skills training but did not focus on workplace skills.
Oshima 2014	**IPS, good fidelity**<br>Four employment specialists, each employed 30 hours per week, formed the IPS unit. The employment specialists received 4 months of training.	**Traditional vocational rehabilitation**<br>Participants received conventional vocational rehabilitation services at the community support centre. The program consisted of prevocational training in various work groups in a simulated environment intended to prepare participants for paid employment.
Poremski 2017	**IPS, good fidelity**<br>Employment specialists were trained. They worked closely with the clinical teams from whose caseloads their clients were drawn.	**Free to seek employment by any means of their choice** Participants were free to seek employment by any means of their choice with some support from their case managers. Available services included training with eventual placement in jobs reserved for people receiving basic social assistance or disability payments. Community-based services for people who were homeless were also available. None of these services were integrated into the clinical teams, or provided time-unlimited personalised support.<br><br>**Note**: Both groups received Housing First, which led to increased housing stability.
Russinova 2018	**Vocational Empowerment Photovoice (high VEP fidelity)**<br>The Vocational Empowerment Photovoice program is a manualised peer-led intervention with a 10-week core component delivered in 2-hr group sessions, which is followed by two booster sessions delivered a month apart after the completion of the core curriculum. Each session combines psychoeducational information, exercises, and group discussions relevant to pursuing employment services and opportunities. Photovoice involves participants using cameras to photograph objects or events in their daily lives that are relevant to a given research topic and generating narratives for these pictures through group discussion. The following elements of the photovoice methodology were embedded in the curriculum: overview of the photovoice process, guidelines about the use of camera and of photography ethics, discussion of the VEP photovoice assignments, taking of pictures relevant to each photovoice assignment, group discussion of selected pictures and writing of corresponding narratives, preparation of photovoice pieces for public display, and discussion of relevant audiences.	**Wait-list control**

Schneider 2016	**IPS plus work-focused counselling intervention** Participants received three to six sessions of work-focused counselling with a psychologist. This intervention was based on psychological practice, including goal-based motivational procedures and cognitive behavioural therapy. This work-focused counselling intervention was designed to enhance the impact of IPS by addressing common obstacles to employment, which are not directly due to symptomatology and not normally the concern of the clinical team. An intervention manual based on a life goals and a problem solving approach was developed and supplemented by self-help materials. It was delivered by a trained psychologist with individual participants. Each received a booklet ('Working Well!') containing information about six topics (anxiety, depression, self-esteem, memory/concentration, stigma and getting on with others), and was asked to choose a maximum of four topics to discuss with the psychologist over up to six sessions lasting about an hour.	**IPS only, range from not IPS to good fidelity**<br>Participants were assigned to an employment specialist who met with them to produce an action plan for employment. Participants continued to meet with their employment specialist as often as they wished.
Smith 2015a	**Virtual reality job interview training**<br>Virtual Reality Job Interview Training is a computer-based intervention developed by SIMmersion LLC (http://www.simmersion.com) to improve interviewing skills for adults with a range of disabilities. See Smith2015b for further information.	**Treatment as usual waitlist control**
Smith 2015b	**Virtual reality job interview training**<br>Virtual Reality Job Interview Training is a software application developed by SIMmersion LLC (www.simmersion.com). Virtual Reality Job Interview Training includes educational content about finding employment, an interactive role-play simulator and integrated feedback. Virtual Reality Job Interview Training allows people with a range of disabilities to repeatedly practice interviews. During each virtual interview, "Molly Porter," a human resources manager at a large department store, asks questions about skills and experiences. Molly selects questions from 1,200 options to tailor each virtual interview on the basis of customisable information, skill level, and responses. Thus, participants can practice until they have gained the skills and confidence to interview for employment.	**Treatment as usual waitlist control**<br>Individuals with Psychiatric Disabilities attended their typical outpatient vocational services for two weeks, which may have included preparations for job interviews using didactic and role-play methods. Veterans with Posttraumatic Stress Disorder attended their usual outpatient services for two weeks, which may have included vocational training.
Tsang 2010	**IPS (Good IPS fidelity)**<br>IPS intervention followed the eight key principles of IPS.<br><br>**Integrated Supported Employment (good IPS fidelity)**<br>The Integrated Supported Employment program combined IPS and work-related social skills training. The main difference with IPS is that it involves 10-session work-related social skills training. The social skill training was provided to participants before obtaining employment. A problem-solving approach was used to help participants handle interpersonal conflicts throughout the follow-up period.<br><br>**Note**: Three registered occupational therapists were employed as employment specialists to implement either the Integrated Supported Employment or IPS protocol. Training for the employment specialists was provided.	**Traditional vocational rehabilitation**<br>Participants received comprehensive vocational assessments and pre-vocational training conducted in the vocational rehabilitation centres. Vocational assessments included work samples, vocational interest exploration, and situational vocational assessments. After the establishment of participants' baseline work performance, pre-vocational training on entry-level job tasks were provided in order to help participants develop specific job skills and work habits. The participants were placed in a sheltered environment in various work groups (e.g. clerical training, computer training, and cleaning training). The aim of the workshop based training was to promote the participants to sheltered workshop or competitive employment. Services were provided by the staff members of service centres providing sheltered vocational training in the community.
Twamley 2012	**IPS (Fair to good IPS fidelity)**	**Traditional vocational rehabilitation**

	IPS participants received manualized supported employment from an employment specialist who had a maximum caseload of 25 participants. Only one employment specialist.	Participants were referred to the Department of Rehabilitation for orientation, intake, and eligibility determination. The conventional vocational rehabilitation was a brokered program for individuals with mental illness. Vocational counsellors had caseloads of 35 participants; additional staff provided job-readiness and prevocational coaching/classes.
Waghorn 2014	**IPS, (Good fidelity)**<br>A full-time employment specialist was trained and employed by the employment service as the sole person delivering vocational services. The employment specialist was co-located at the mental health service four days a week.	**Non-integrated forms of supported employment (Fair IPS fidelity)**<br>Mental health case managers referred participants to other disability employment services in the local area. Regular communication with the employment specialist was then encouraged to facilitate participant engagement and to monitor progress.
Yamaguchi 2017	**Cognitive remediation and supported employment**<br>Participants received cognitive remediation in psychiatric day-care or community employment service agencies during the first 3 or 4 months. Psychiatric day-care provides social skills, recreation and a daytime place to stay. However it does not focus on employment services, acute care to prevent hospital admission, or care for people with severe mental illness. Community employment service agencies provide a group-based work readiness training for people with mental illness before they undergo a job search, and are not integrated with medical services. The programme involved two CogPack sessions per week over 12 weeks (24 sessions in total). Tasks were related to attention, concentration, psycho-motor speed, learning, memory and executive functions. In addition, participants had 1-hour verbal group sessions each week following one of CogPack sessions (total: 12 sessions). They discussed the importance of cognitive skills, performing activities of daily living and the development of compensatory strategies for managing persistent cognitive problems, to bridge individuals' learning outcomes in the computer-based cognitive remediation programme and social/employment skills in their lives. Supported employment services were provided to participants after completion of cognitive remediation. In this study, these services partly incorporated the service principals of the IPS model. The supported employment programme was not IPS model due to unique employment laws in Japan, and the IPS fidelity scale was not used. During the study period, the staff received the 1-day training for five times to learn skills for cognitive remediation and supported employment.	**Traditional vocational rehabilitation**<br>The usual employment services currently used in Japan are based on the brokerage care management model. The care managers in the hospitals met the participants at least once a month and engaged with them and with community facilities that provided the traditional employment services. Most such facilities in Japan employ the train-then-place/step-wise model that attempts to improve work-readiness in people with mental illness and supplies training and simple tasks before actual job hunting.
**Studies with people with autism**		
**Study**	**Interventions**	**Control/ Comparator**
Wehman 2014, Wehman 2017	**Project SEARCH plus ASD supports**<br>Project SEARCH is an intensive job training program where youth with developmental disabilities in their final year of secondary school are placed in a large community business (e.g. hospital, government complex or banking centre). The program comprised an internship portion with supported employment and a classroom portion with educational supports. Student logged approximately 720 hours of internship time and 180 hours of classroom time at the business for a total of 900 hours in the business setting. Students rotate through three 10–12 week internships within the business while receiving supported employment. The four phases of supported employment include: (1) job seeker profile, (2) job development, (3) job site training, and (4) long-term supports. Supported employment is a highly individualised	**High school special education services as usual**<br>Regular high school special education program as identified in their individualised education programs. These plans typically include an array of related services including special education, one-on-one instruction, and behavior management from a paraprofessional assistant, speech and language therapy, occupational therapy, social skills training, and limited vocational training. Students also had access to vocational rehabilitation services like those in the treatment group.

	approach that allows for time-unlimited services. Additional supports for individuals with ASD were added to Project SEARCH. The applied behavior analytic techniques used included the use of scored task analyses for teaching multistep tasks, structured repeated trials for discrete tasks, behavioural rehearsal for specific social skills, visual and self-directed prompting procedures for transitioning, self-management procedures for behavioural challenges, and reinforcement for appropriate behavior. Participants with ASD were provided assistance to understand common work statements in behavioural terms. When a student displayed problem behavior, a functional behavior assessment was completed and behavior intervention plans were implemented to address the behavior. Customised employment was used to analyse workplace tasks and identify which tasks were most suited to the strengths of participants with autism. Staff members received additional training in the needs of transition-aged youth with ASD. The staffing ratio was two/ two-and-a-half interns to one staff member.	
Wehman 2020	**Project SEARCH plus ASD Supports**<br>Project SEARCH is a job training program where students with autism spend their final year of secondary school in a combination classroom and internship program located in a large community business. Participants rotated through three 10–12 week internships to learn marketable skills. Participants received their entire school week in community based employment training. Hence, participants received 35 hours of community based employment training a week.	**High school special education services as usual**<br>Students attended their assigned high school and received the services, accommodations, and modifications stipulated in their individualised education plans. In addition to these school based services, all but one control group participant received some community based employment training. All educational services were provided by school district staff.
Whittenburg 2020	**Project SEARCH plus ASD Supports**<br>Project SEARCH is a job training program, where students with developmental disabilities spend their final year of secondary school undertaking internships and participating in daily classroom-based instruction on employability skills. Project SEARCH participants rotate through three 10- to 12-week internships in large community businesses (e.g. hospital, government complex or banking centre) based on their preferences and strengths in order to learn marketable skills, social communication, and adaptive behavior for employment. Autism-specific supports include: (1) onsite, intensive, systematic instruction using applied behavioural analysis principles; (2) onsite support and consultation from a behaviour/autism specialist; and (3) intensive staff training in ASD and the Project SEARCH Model.	**Waitlist high school special education services as usual**<br>Students received public school-based, special education transition services including participation in hands-on work training in community and/or school settings and classroom-based instruction on functional and academic skills.

ASD: Autism Spectrum Disorder

IPS: Individual Placement and Support; Note: IPS is described in the first instance only but any study-specific information about intervention has been included where provided.
